# Comprehensive analyses of the *BES1* gene family in *Brassica napus* and examination of their evolutionary pattern in representative species

**DOI:** 10.1186/s12864-018-4744-4

**Published:** 2018-05-09

**Authors:** Xiaoming Song, Xiao Ma, Chunjin Li, Jingjing Hu, Qihang Yang, Tong Wang, Li Wang, Jinpeng Wang, Di Guo, Weina Ge, Zhenyi Wang, Miaomiao Li, Qiumei Wang, Tianzeng Ren, Shuyan Feng, Lixia Wang, Weimeng Zhang, Xiyin Wang

**Affiliations:** 1 Center of Genomics and Computational Biology, College of Life Sciences, North China University of Science and Technology, Tangshan, 063210 Hebei China; 2 Library, North China University of Science and Technology, Tangshan, 063210 Hebei China

**Keywords:** *BES1* gene family, Evolutionary trajectory, Polyploid, Positive selection, Expression pattern, *B. napus*

## Abstract

**Background:**

The *BES1* gene family, an important class of plant-specific transcription factors, play key roles in the BR signal pathway in plants, regulating various development processes. Until now, there has been no comprehensive analysis of the *BES1* gene family in *Brassica napus*, and a cross-genome exploration of their origin, copy number changes, and functional innovation in plants was also not available.

**Results:**

We identified 28 *BES1* genes in *B. napus* from its two subgenomes (AA and CC). We found that 71.43% of them were duplicated in the tetraploidization, and their gene expression showed a prominent subgenome bias in the roots. Additionally, we identified 104 *BES1* genes in another 18 representative angiosperms and performed a comparative analysis with *B. napus*, including evolutionary trajectory, gene duplication, positive selection, and expression pattern. Exploiting the available genome datasets, we performed a large-scale analysis across plants and algae suggested that the *BES1* gene family could have originated from group F, expanding to form other groups (A to E) by duplicating or alternatively deleting some domains. We detected an additional domain containing M4 to M8 in exclusively groups F1 and F2. We found evidence that whole-genome duplication (WGD) contributed the most to the expansion of this gene family among examined dicots, while dispersed duplication contributed the most to expansion in certain monocots. Moreover, we inferred that positive selection might have occurred on major phylogenetic nodes during the evolution of plants.

**Conclusions:**

Grossly, a cross-genome comparative analysis of the *BES1* genes in *B. napus* and other species sheds light on understanding its copy number expansion, natural selection, and functional innovation.

**Electronic supplementary material:**

The online version of this article (10.1186/s12864-018-4744-4) contains supplementary material, which is available to authorized users.

## Background

Plants often suffer a series of biotic and abiotic stresses, causing a decrease in yield and quality. Transcription factors, which can bind to specific sequences, play important and critical roles in plant growth and resistance to various stresses [1]. The *BES1* (*BRI1-EMS-SUPPRESSOR1*) gene family is a novel class of plant-specific transcription factors that regulate BR-responsive genes [2]. Brassinosteroids (BRs) are a class of plant steroid hormones that regulate various development processes, such as leaf development, stem elongation, pollen tube growth, xylem cell differentiation, senescence, and photomorphogenesis [3–5]. Plants are often protected by BRs from all kinds of environmental stresses, including low- and high-temperatures, salinity, drought, injury, pathogens, and insect attack [6–9].

The *BES1* gene family plays key roles in the BR signalling pathway. BES1 binds to the promoter region of *SAUR-AC1*, which has become a molecular tool to dissect brassinosteroid and auxin responses [2, 10]. In addition, BES1 interacts with a bHLH protein BIM1 (BES1-interacting Myc-like1) to synergistically bind to E-box sequences that are present in many BR-induced gene promoters [2, 11]. In *A. thaliana*, several members of the *BES1* gene family have been identified, such as *BES1*, *BZR1*, and *BEH1–4*. These transcription factors have a gene function that is redundant in the BR signalling pathway [2]. The *BES1* genes contain an NLS (Nuclear localization signal), followed by a highly conserved amino-terminal domain (N), phosphorylation domain (P), a PEST motif, and a carboxyl-terminal domain (C). The central P domain of *BES1* genes is the target of BIN2 kinase, and the PEST motif is implicated in protein degradation [2, 12].

There are few prior studies on the evolution of the *BES1* gene family. Recent studies have found that the *BES1* locus in *A. thaliana* encodes two isoforms, long and short *BES1* (*BES1-L* and *BES1-S*). Among them, *BES1-L* has an additional 22 amino acids in the N terminal, and it has a stronger biological function than *BES1-S*. It can promote the nuclear localization of BZR1 and BES1-S [13]. *BES1-L* is an important isoform in the BR signalling pathway, and it uniquely exists in the majority of *A. thaliana* ecotypes. *BES1-L* is a more recently evolved isoform and may have contributed to the expansion and evolution of *A. thaliana* [13]. *BES1* shows relaxed selection compared to *BZR1*, hallmark of sub- and neofunctionalization, and dynamic HSP90 client status across independent evolutionary paths [14]. By ChIP-chip and gene expression analyses, a comprehensive transcriptional network for *BES1*-regulated genes was constructed, which contained 1609 putative *BES1* target genes [15]. For a member of the *BES1* gene family, the phylogenetic tree indicates that angiosperm *BZR1*-like genes form two groups, *BZR1* and *BZR2*/3/4. However, only one *BZR1*-like gene was detected in the basal extant angiosperm (*A. trichopoda*). These two groups may be generated by a gene duplication event before the diversification of extant angiosperms, while *A. trichopoda* lost the *BZR1* member during its evolution [16]. Previously, we also conducted a genome-wide analysis of the *BES1* gene family in Chinese cabbage, and explored its structural and functional changes in the process of evolution [17].

In that it is pivotal in its importance to plant growth, the *BES1* gene family has been identified and analysed in some plants, including *A. thaliana* [2], *O. sativa* [18], and *B. rapa* [17]. However, in *B. napus*, there is a lack of studies on the function and structure of the evolution of the *BES1* gene family. *B. napus* is an important oilseed crop grown worldwide as a member of the genus Brassica. *B. napus* (AACC genome), an allopolyploid, originated from hybridization of *B. rapa* (AA genome) and *B. oleracea* (CC genome) [19]. The availability of these Brassica genomes, together with those of a model species, provided an opportunity to understand the formation and evolution of the *BES1* gene family.

The aims of this study were to: (i) identify and characterize the *BES1* gene family in *B. napus* and 18 other species to understand their evolutionary pattern across plant species; (ii) to characterize their phylogenetic relationships, origin and evolution, and infer duplicated genes; (iii) by inferring gene collinearity, to find how polyploidization has contributed to the evolution of this gene family; and (iv) to explore expression patterns of *BES1* genes in two tissues based on the transcriptome and microarray data. This comprehensive analyses contributes to our understanding of the functional innovation of this important gene family, as well as the effect of gene loss, retention, and expansion, especially due to recursive polyploidization.

## Methods

### Retrieval of genome sequences

**Fig. 1:**
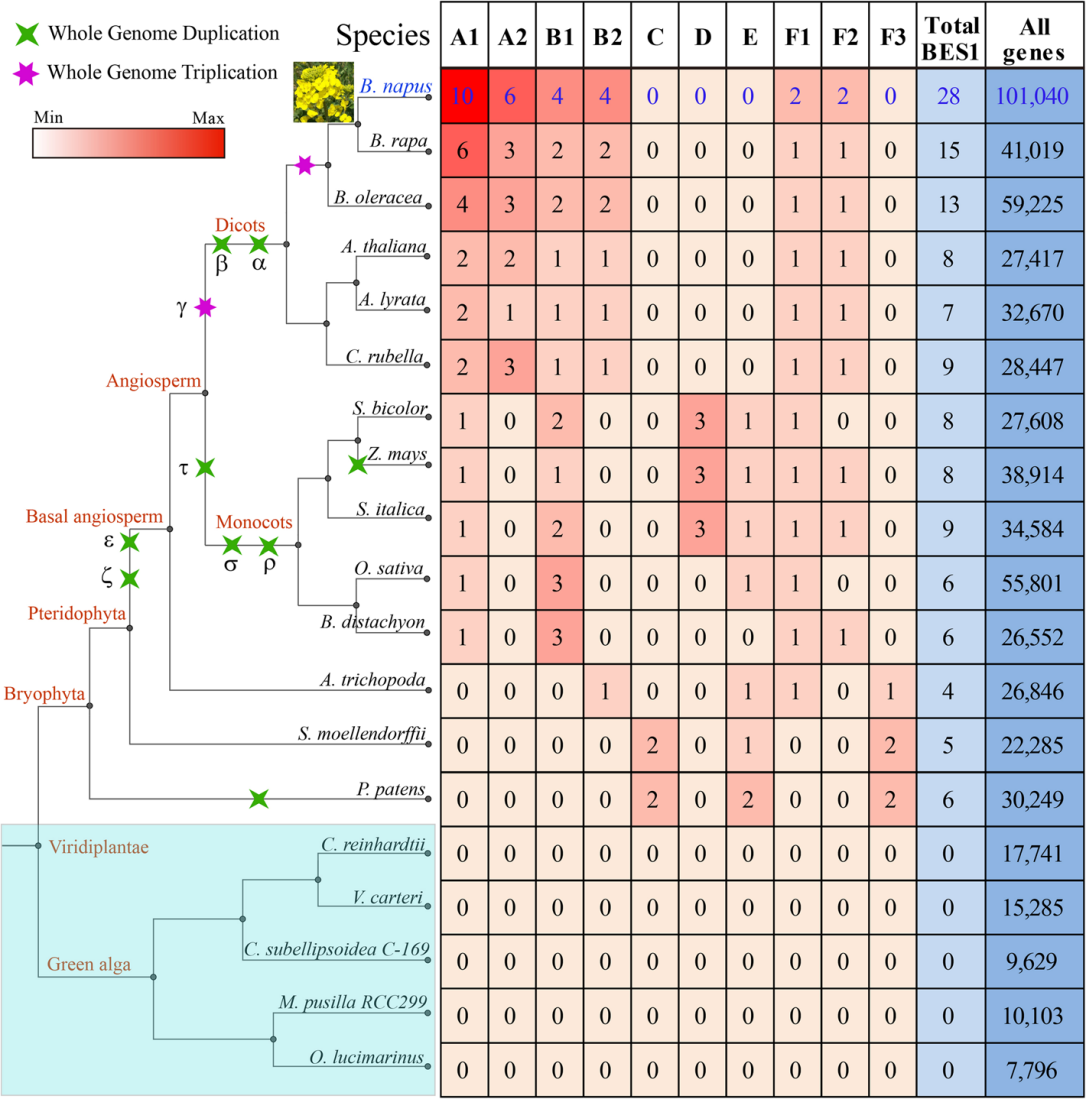
The number and classification of *BES1* genes in *B. napus* and the other 18 species used in this study. Information regarding genome duplication was obtained from the Plant Genome Duplication Database (PGDD)

The *B. napus* and 18 representative genome sequences were used for comparative analyses. These species contained 6 dicots, 5 monocots, 1 basal angiosperm, 1 Pteridophyta, 1 Bryophyta, and 5 green algae (Fig. 1). The genome sequences of *B. napus* were downloaded from the Genoscope genome database [19]. The *A. thaliana* sequences were downloaded from TAIR, and the rice sequences were downloaded from RGAP [20]. The *B. rapa* sequences were from BRAD [21] and the *B. oleracea* sequences were from EMBL (http://www.ebi.ac.uk/). The *A. trichopoda* sequences were downloaded from the Amborella Genome Database [22]. The sequences of the other species were downloaded from phytozome [23].

Identification and characterization of the *BES1* gene family.

The Pfam database (version 31.0) was used to identify *BES1* genes from all protein sequences with a threshold of e-value <1e-5 [24]. *BES1* genes have the typical BES1_N domain (PF05687.9). The retrieved *BES1* candidates were further verified by using the SMART and Conserved Domain Database with default parameters [25, 26]. To eliminate possible pseudogenes, sequences with < 150 amino acids were removed for further analysis. MEME (version 4.12.0) was used to search for conserved motifs, and the number of motifs was set as 10 [27]. The protein structure was predicted using the Swiss-model database [28]. The interaction networks of *A. thaliana BES1* genes were constructed using the STRING database (http://string-db.org). The RNA-seq data was used to analyse *BES1* gene expression in leaves and roots in *B. napus* [19]. This dataset contained three replicates, and the RPKM value was log10 transformed. Expression data of *A. thaliana* genes were obtained from the BAR database (http://bar.utoronto.ca/) [29, 30]. The expression analysis of *BES1* genes under heat, cold and salt treatments by qRT-PCR was conducted according to our previously reported methods [31, 32].

### Sequence alignment and phylogenetic analyses

Multiple sequence alignment was performed using MUSCLE with default parameters [33]. Based on alignment, we generated a phylogeny using previously reported methods [34]. Phylogenetic analyses were conducted using three methods: Neighbour-joining (NJ), Maximum Likelihood (ML), and Bayesian. NJ trees were constructed using MEGA 6.0 with 1000 bootstrap and a Poisson correction model [35]. The MrBayes v3.2.6 software was employed to construct Bayesian trees using the fixed (Jones) model for amino acid substitutions that was run for 4 × 10^6^ generations, with 6 Markov chains, sampled every 1 × 10^4^ generations [36]. PhyML 3.0 was employed to construct ML trees, with the Jones, Taylor and Thorton (JTT) model, and 100 nonparametric bootstrap replicates [37]. The reconstructed *BES1* gene trees were compared with real species trees by lineage using Notung 2.9 software with default parameters [38].

### Orthologs, paralogs, duplicated type, and collinear blocks identification

Orthologous and paralogous *BES1* genes were identified using OrthoMCl (v2.0, e-value: 1e-5), and then we counted the orthologous gene pairs for each *A. thaliana BES1* gene manually [39]. Their relationships were constructed using Cytoscape (v3.6.0) [40]. First, whole-genome protein sequences from all species were searched against themselves using BLASTP with an E-value of 1 × 10^− 5^. MCScanX v1.0 (−k 50, −s 5, −m 25) was then used to detect the duplicated type and collinear blocks according to a previous report [41]. Then, we extracted the *BES1* genes located in the collinear blocks by Perl scripts.

### Selective pressure detection in each group

To estimate the divergence time between collinear *BES1* gene pairs, alignment of protein sequences was performed using ClustalW2.0, and then translated into CDS alignment. We applied likelihood ratio tests of positive selection based on ML methods and codon substitution models. Based on reported methods [42, 43], to find evolutionary traces of selective pressures, we analysed each group to infer ω (the ratio of nonsynonymous to synonymous distances) using Codeml implemented from PAML4.9 [44, 45]. We employed a complete deletion method when analysing alignments with gaps, and eliminated sequences that contained 40% of their length or more as InDels. We detected variation in ω among sites by employing a likelihood ratio test between the M0 and M1 and M7 and M8 models.

## Results

### Identification and characterization of the *BES1* gene family

We systematically identified *BES1* genes by searching Pfam database in *B. napus* and 18 other representative species, including 5 dicots (*B. rapa*, *B. oleracea*, *A. thaliana*, *A. lyrata*, and *C. rubella*), 5 monocots (*S. bicolor*, *Z. mays*, *S. italica*, *O. sativa*, and *B. distachyon*), 1 basal angiosperm (*A. trichopoda*), 1 Pteridophyta (*S. moellendorffii*), 1 Bryophyta (*P. patens*), and 5 green algae species (Fig. 1). After filters, a total of 132 *BES1* genes were identified among all species. Allotetraploid plant *B. napus* contained the most *BES1* genes (28), being just the addition of its two diploid progenitors *B. rapa* (15) and *B. oleracea* (13) (Fig. 1). Generally, there were more *BES1* genes in diploid *Brassica* genomes than others due to their shared genome duplication event. No *BES1* gene was identified in the five green algae species. This result indicated that the *BES1* genes were subjected to large-scale expansion in higher plants.

### Phylogenetic and classification analysis of *BES1* genes

**Fig. 2:**
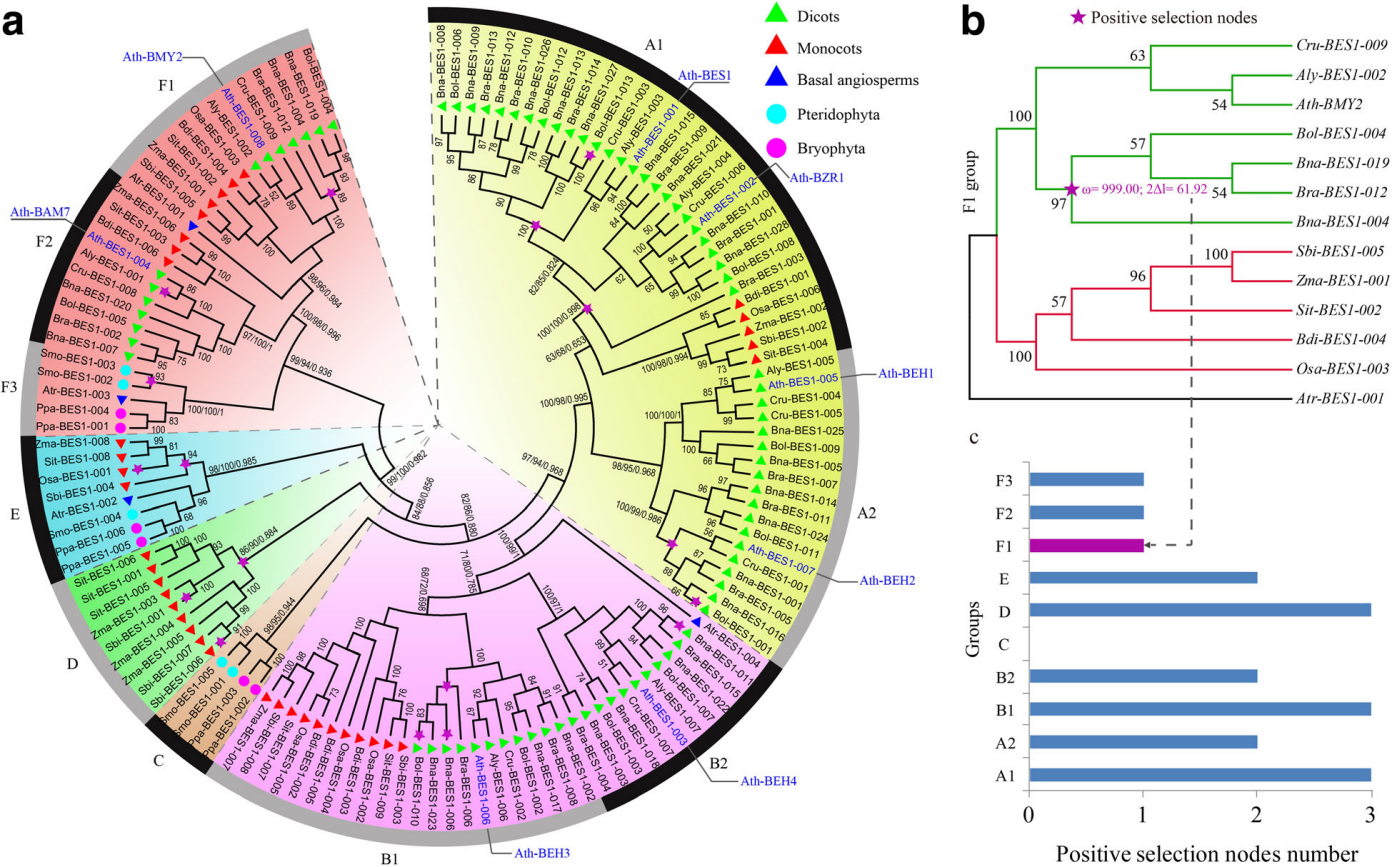
Phylogenetic relationship and positive selection analyses of the *BES1* gene family. **a** Phylogenetic tree topology was generated via MEGA6.0. For the major nodes, neighbour-joining (NJ) and maximum-likelihood (ML) bootstrap values above 50% are shown, followed by Bayesian posterior probability values (> 0.6) by MrBayes. The A to F indicate the groups obtained by the bootstrap values and phylogenetic topology. **b** Positive selection analyses of groups F1 in representative species. The ω on the clades is the dn/ds value under the M8 model of codeml. **c** The positive selection nodes number in each group in the representative species

To conduct the classification of *BES1* genes, we constructed a phylogenetic tree using all of the examined species by MEGA 6.0. The 132 *BES1* genes of the 14 species were clearly grouped into six groups (A to F) according to the bootstrap values and phylogenetic topology (Fig. 2a). Furthermore, groups A and B were divided into 2 distinct subgroups (A1 and A2, B1 and B2), respectively. The group F was further divided into 3 subgroups corresponding to F1 to F3. In *P. patens* and *S. moellendorffii*, all *BES1* genes were assigned into C, E, and F3 groups (Figs 1, 2a). However, there was no *BES1* gene detected in group C among the angiosperm species, indicating that the genes of group C might have experienced an evolutionary divergence in structure or function.

Among *B. napus* and other 5 dicots, we found that more than 70% of genes belonged to groups A and B, and there was no *BES1* gene in groups C, D, E, and F3 (Fig. 1). Furthermore, the results showed that the expansion of *BES1* genes in 3 *Brassica* species mainly occurred in groups A and B due to their genome duplication events. In the 5 monocots species, there was no gene in groups A2, B2, C, and F3 (Fig. 1). Interestingly, we found that groups D and E contained *BES1* genes in most of the monocots but not in the dicots. In addition, we noted that *S. bicolor*, *Z. mays*, and *S. italica* contained 3 genes in group D, while there was no gene in this group for *O. sativa* and *B. distachyon*. These results indicated that there have been lineage-specific expansion of *BES1* genes after the divergence of dicots and monocots.

### Positive selection analyses for the *BES1* gene family

Strong positive selection was observed for the major nodes on the phylogenetic tree, which possibly contributed to the divergence of higher plants. To uncover whether and when natural selection had acted on the evolution of the *BES1* gene family, we performed selection pressure analyses in each group using PAML program. Taking the F1 group as an example, 1 significantly nonsynonymous vs synonymous substitution (ω = 999.00, 2Δl = 61.92) was detected, showing a strong positive selection after the divergence of *Brassica* and other species (Fig. 2b). This branch contained 4 *Brassica* genes, such as *Bol-BES1–004*, *Bna-BES1–019*, *Bra-BES1–012*, and *Bna-BES1–004*.

Furthermore, we also detected positive selection for other groups (Additional file 1: Figure S1). The results showed that there were more positive selection nodes in groups A1, B1, and D than in the other groups, and the number reached 3 (Fig. 2c). In groups A2, B2, and E, 2 positive selection nodes were detected, and there was no positive selection node in group C. Generally, we found there were more positive selection nodes in dicots (12, groups A, B, F1, and F2), followed by monocots (5, groups D and E). This result indicated that *BES1* genes in dicots underwent more positive selection than those in other lineages.

### Conserved motif analysis of *BES1* genes exploring their origin and evolutionary pattern

**Fig. 3:**
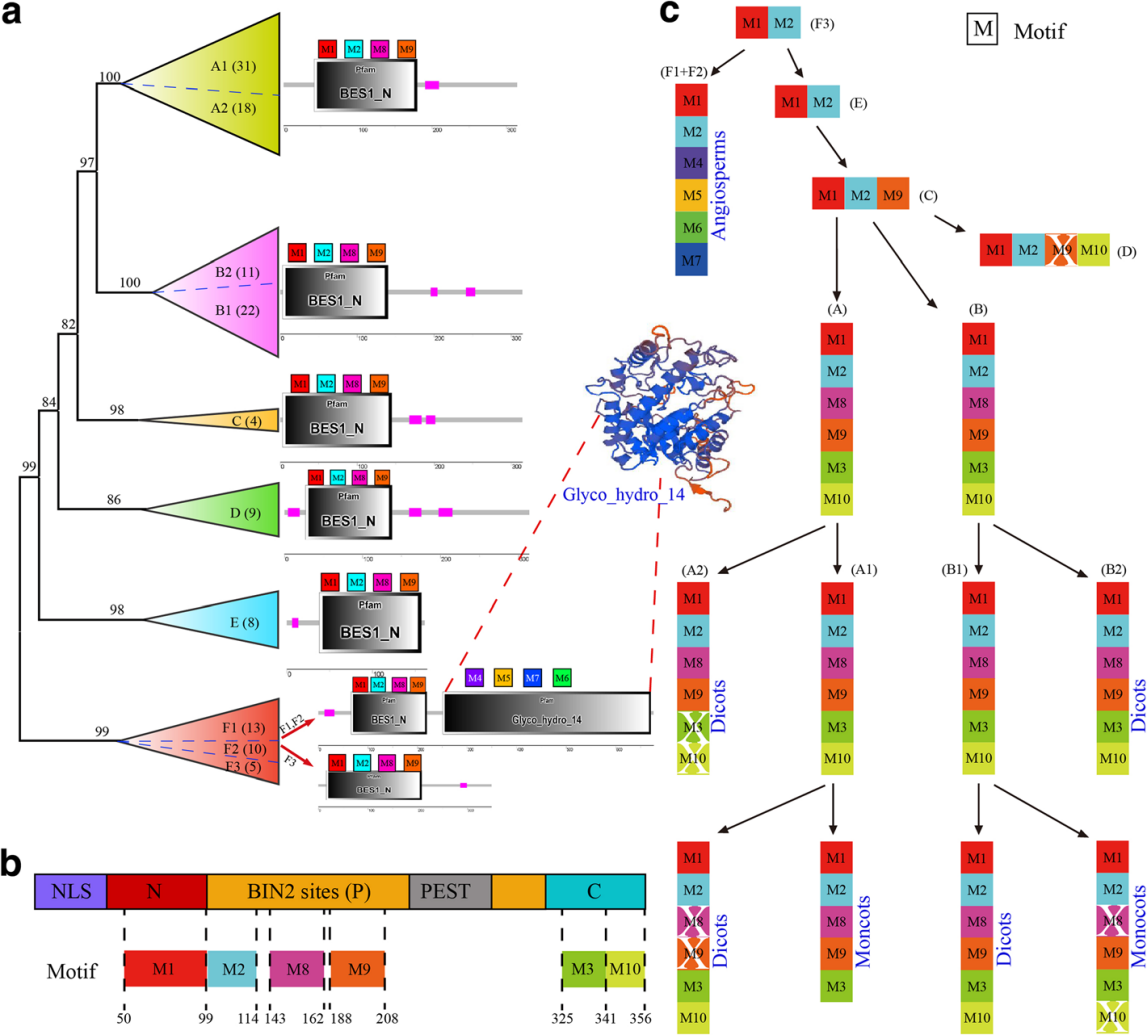
The converted motif and evolutionary trajectories analyses of the *BES1* gene family in plants. **a** The motif 1 to motif 10 (M1 to M10) of the *BES1* gene family for each group. **b** The correspondence relationship between schematic structures of *BES1* and motifs. **c** The major evolutionary trajectories of the *BES1* gene family. The white X indicated the motif was lost in some genes

To further explore the origin and evolutionary pattern of *BES1* genes, the most conserved 10 motifs (M) were detected by MEME program (Fig. 3a, Additional file 2: Figure S2). The M1 was located at the amino-terminal domain (N), and M2, M8, and M9 constituted BIN2 phosphorylation sites (P), and M3, M10 belonged to the carboxyl domain (C) (Fig. 3b). All genes had M1 and M2, while the other 8 motifs existed in some but not all groups (Additional file 2: Figure S3).

We found specific preservation and expansion of motifs in different plant lineages (Fig. 3c). Among F subgroups, F3 contained 5 genes, including 2 *P. patens*, 2 *S. moellendorffii*, and 1 *A. trichopoda* genes, while subgroups F1 and F2 only contained angiosperm genes. Interestingly, we found subgroups F1 and F2 contained M4 to M7, which were absent in F3 and other groups. For group E, only M1 and M2 were detected, and this group did not contain dicot genes. For group C, M1, M2, and M9 were identified, which only contained 2 *P. patens*, and 2 *S. moellendorffii* genes. Group D was specific for monocots, and contained M1, M2, M9, and M10, while M9 might have lost some genes. Two main groups, A and B, existed in most angiosperm species. Most genes in these two groups contained M1, M2, M8, M9, M3, and M10.

For genes in groups F1 and F2, we detected an additional domain, glyco_hydro_14, being contained in M4 to M8 (Additional file 2: Figure S3). To explore the sequence divergence of *BES1* genes among F1, F2 and F3, we conducted sequence alignments using all genes belonging to the F group (Additional file 2: Figure S4). The results showed that the first 70 amino acids of the *BES1_N* domain were very conserved among the three subgroups. We found 12, 36, 40, and 45 amino acids in the *BES1_N* domain were the same in the F1 and F2 subgroups, which were different from the F3 subgroup. For the Glyco_hydro_14 domain, we found that it was conserved between the F1 and F2 subgroups, while it was divergent or had lost parts in the F3 subgroup (Additional file 2: Figure S4). We further conducted protein structure analyses using the Swiss-model database. We found that its homology model was 1fa2.1.A, with a similarity of up to 46.59% (Additional file 2: Figure S5a). Further assessment also showed that this predicted model had high quality and accuracy (Additional file 2: Figure S5b–d).

Identification of orthologous and paralogous *BES1* genes between *B. napus* and other species.

**Fig. 4:**
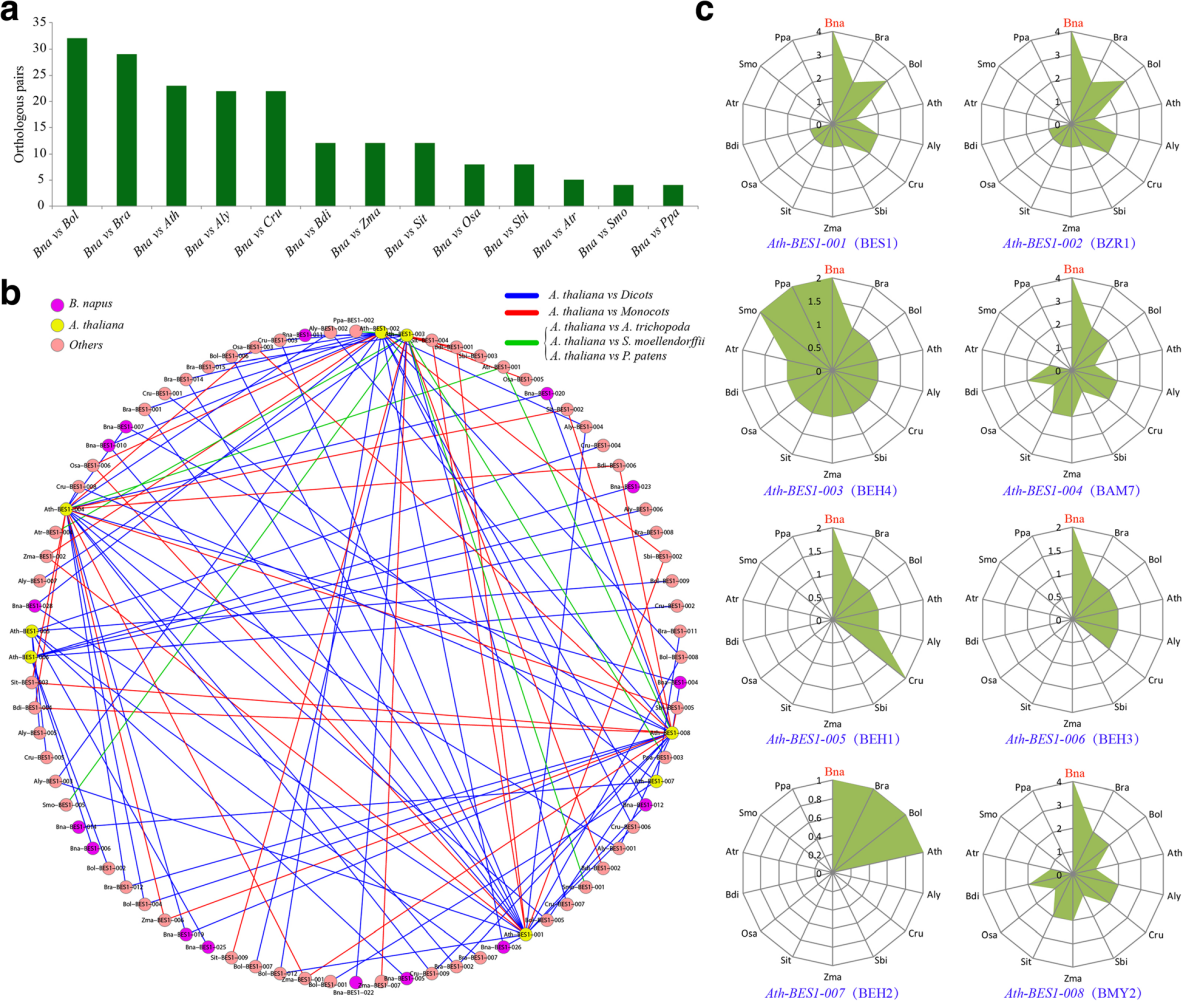
The analyses of orthologous *BES1* genes across species. **a** The orthologous *BES1* gene pairs between *B. napus* and other examined species. **a** The interaction network constructed using the orthologous gene pairs between *A. thaliana* and other species. **b** The number of orthologous genes in each species with 8 *A. thaliana BES1* genes. The abbreviations represent the species as follows: *Bna*, *B. napus*; *Bra*, *B. rapa*; *Bol*, *B. oleracea*; *Ath*, *A. thaliana*; *Aly*, *A. lyrata*; *Cru*, *C. rubella*; *Sbi*, *S. bicolor*; *Zma*, *Z. mays*; *Sit*, *S. italica*; *Osa*, *O. sativa*; *Bdi*, *B. distachyon*; *Atr*, *A. trichopoda*; *Smo*, *S. moellendorffii*; *Ppa*, *P. patens*

We identified orthologous gene pairs between *B. napus*, *A. thaliana*, *O. sativa*, *A. trichopoda*, *S. moellendorffii*, *P. patens* and other species, respectively using the OrthoMCL program (Additional file 3: Table S1). There were more orthologous pairs between *B. napus* and *B. oleracea* (32), followed by *B. rapa* (29) and *A. thaliana* (23) (Fig. 4a). Taking the orthologous genes between *A. thaliana* and other species as an example, we constructed an interaction network (Fig. 4b). There were more orthologous gene pairs for *Ath-BES1*, *Ath-BZR1*, *Ath-BAM7*, *Ath-BEH3*, and *Ath-BMY2* in *A. thaliana* versus *B. napus* than versus others (Fig. 4c). For *Ath-BEH2*, only one orthologous gene pair was identified for *A. thaliana* versus each *Brassica* species, while there was no orthologous gene between *A. thaliana* and others. In *O. sativa*, there were more orthologous genes between *O. sativa* and monocots than other comparisons (Additional file 2: Figure S6a). For *Osa-BES1–003*, 4 orthologous gene pairs were detected between *O. sativa* and *B. napus*, which was more than for other comparisons (Additional file 2: Figure S6b). Furthermore, orthologous gene pairs were also detected between *A. trichopoda*, *S. moellendorffii*, *P. patens,* and other species (Additional file 2: Figure S7–9).

Whole-genome duplication (WGD) may have contributed to the expansion of the gene family. Additionally, 15 paralogous *BES1* gene pairs were identified in *B. napus*, which was far more than in *A. thaliana* and other plants (Additional file 2: Figure S10, Additional file 3: Additional file 3: Table S2). This might be a result of additional WGDs after its split from *A. thaliana*. In addition, we found that 4 *BES1* genes in *B. napus* had more than 1 paralogous gene, including *Bna-BES1–007*, *Bna-BES1–004*, *Bna-BES1–019*, and *Bna-BES1–020* (Additional file 3: Figure S10). In *A. trichopoda*, there were no paralogous *BES1* genes. Therefore, the number of paralogous information could partly reflect species genome duplication.

### Duplicated type identification and synteny analyses of *B. napus* and other species

Various gene duplications may have contributed to the expansion of the gene family. We found evidence that WGD likely contributed the most to the expansion of this gene family in many plant lineages. We examined 5 types of gene duplications: singleton, dispersed, proximal, tandem, and WGD or segmental duplication by MCScanX program (Table 1, Additional file 3: Table S3). The percentage of WGD was 75.0% in *B. napus*, *B. rapa* (80.0%), *B. oleracea* (84.6%), and *A. thaliana* (62.5%) (Table 1). However, WGD or segmental duplication contributed the most to gene expansion only in *O. sativa* (50.0%) and *B. distachyon* (66.7%). In the other three monocots, the dispersed duplication contributed the most to gene expansion. For *A. trichopoda* and *S. moellendorffii*, all *BES1* genes belonged to the dispersed duplication. Actually, by checking gene collinearity within a genome, we found that more than 55.0% of *BES1* genes in *B. napus* and the other 5 dicots were located in collinear blocks, showing their duplication during polyploidization (Additional file 3: Table S4). Especially for the 3 *Brassica* species, more than 75% of *BES1* genes were located in collinear blocks. A percentage of 37.5, 37.5, and 33.3% of *BES1* genes were located in collinear blocks for *S. bicolor*, *Z. mays*, and *S. italica*, respectively. Furthermore, we found that the percentage of *BES1* genes located in the collinear blocks was larger than the average genome-wide level for all examined species except *A. trichopoda* and *S. moellendorffii* (Additional file 3: Table S4). The percentage for *BES1* genes in colinearity was significantly larger than the average genome-wide level by χ^2^ test, and *P-values* were 6.69E-18 and 1.21E-17 for dicots and monocots, respectively.

**Table 1:** The identification of duplicated type for *BES1* genes and all genes in *B. napus* and other representative species

Species	Singleton		Dispersed		Proximal		Tandem		WGD or segmental		Total	
	Genome	*BES1*	Genome	*BES1*	Genome	*BES1*	Genome	*BES1*	Genome	*BES1*	Genome	*BES1*
*B. napus*	7768	0	26,907	7	2428	0	2708	0	61,229	21	101,040	28
*B. rapa*	3666	0	10,622	3	873	0	2369	0	23,489	12	41,019	15
*B. oleracea*	4807	0	25,232	2	2515	0	2523	0	24,148	11	59,225	13
*A. thaliana*	5156	0	10,670	3	1046	0	3026	0	7519	5	27,417	8
*A. lyrata*	5585	0	14,735	3	1936	0	3037	0	7376	4	32,670	7
*C. rubella*	3792	0	10,428	3	1144	0	6142	1	6941	5	28,447	9
*S. bicolor*	4756	0	12,434	4	1295	1	3684	0	5439	3	27,608	8
*Z. mays*	7468	1	16,126	4	1427	0	2182	0	11,711	3	38,914	8
*S. italica*	7408	0	14,785	4	2183	0	4365	2	5843	3	34,584	9
*O. sativa*	12,368	1	30,411	2	3761	0	3533	0	5728	3	55,801	6
*B. distachyon*	4857	0	12,877	2	1280	0	2822	0	4716	4	26,552	6
*A. trichopoda*	8608	0	13,985	4	1849	0	2195	0	209	0	26,846	4
*S. moellendorffii*	4164	0	10,621	5	2094	0	1143	0	4263	0	22,285	5
*P. patens*	9966	0	14,241	4	1083	0	979	0	3980	2	30,249	6

### Differential *BES1* gene losses and duplications during evolution

**Fig. 5:**
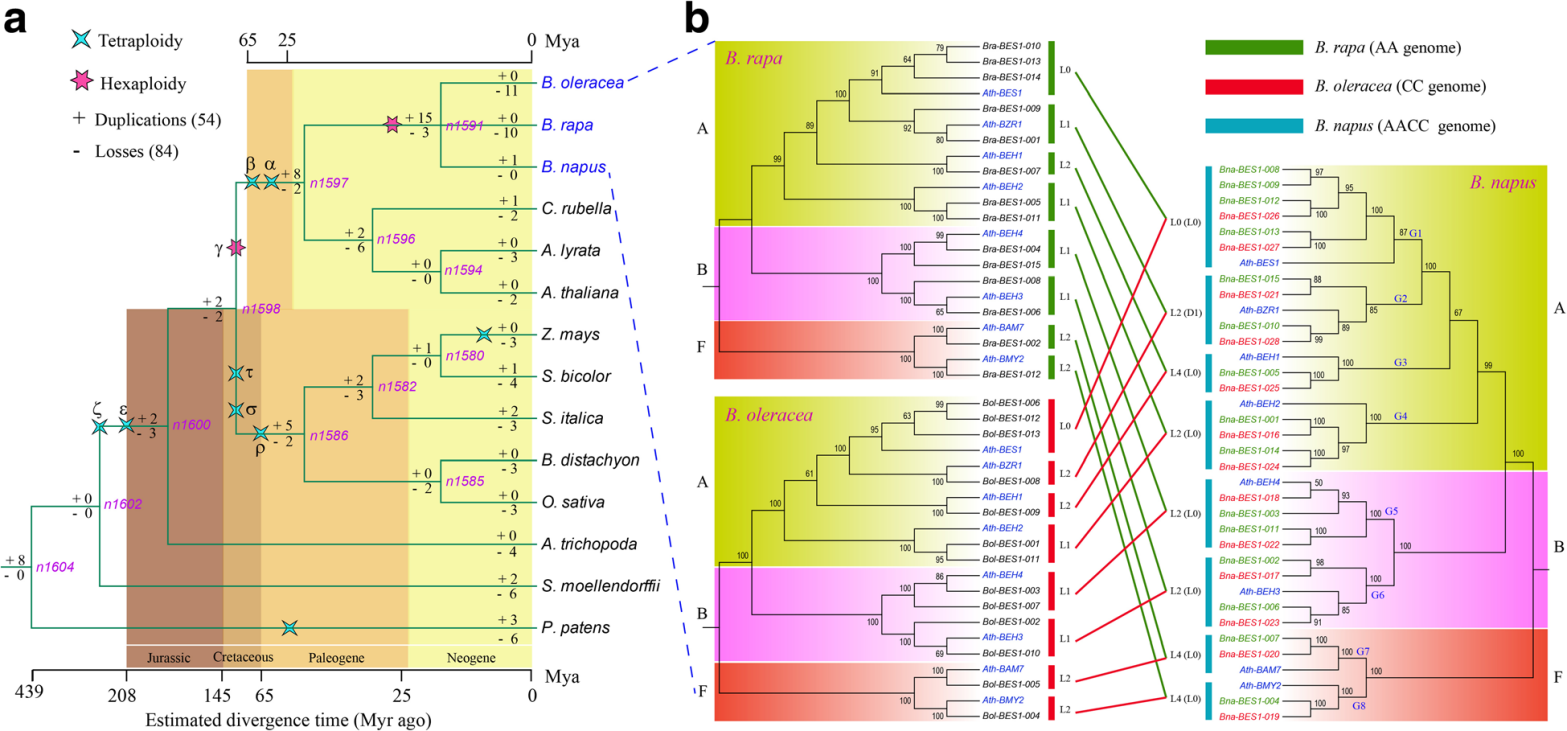
The duplication or losses analyses of *BES1* genes. **a** Number variations of *BES1* genes at different stages of plant evolution. Differential gene losses and gains are indicated by numbers with - or + on each branch. WGD events are indicated with a quadrilateral or hexagon, respectively. **b** The duplication or losses analyses of *BES1* genes in *A. thaliana* and 3 *Brassica* species

Expansion of *BES1* genes in WGDs was also accompanied by gene losses. To clarify the evolution of the *BES1* gene family, we conducted the gene loss and duplication analyses using Notung [46]. We obtained the number variations of *BES1* genes at different stages of evolution according to the reconstructed phylogenies (Fig. 5a, Additional file 2: Figure S11). In the lineage leading to the common ancestor of *P. patens*, *S. moellendorffii*, and angiosperms, 8 ancestral genes were duplicated, which occurred 439 Mya, while no gene was lost. In the lineage of the common ancestor for *A. trichopoda* and angiosperms, 2 ancestral genes were duplicated, and 3 genes were lost, which occurred during the Jurassic period. In the lineage leading to the common ancestor of dicots and monocots, 2 ancestral *BES1* genes were duplicated and lost, respectively (Fig. 5a). For the branch of the common ancestor of *B. napus* and 5 dicots, 8 ancestral genes were duplicated, and 2 genes were lost. Since the divergence, the *A. thaliana* lineage lost 2 genes and there were no duplications. For 3 *Brassica* species, 15 ancestral genes were duplicated, and 3 genes were lost in the branch of their common ancestor. The *B. oleracea* and *B. rapa* lineage lost 11 and 10 genes, respectively, and no duplicated genes were detected. However, the *B. napus* lineage had no gene losses, and only 1 duplicated gene was detected.

For the 5 monocots, 5 ancestral *BES1* genes were duplicated, and 2 genes were lost in their lineage of the common ancestor (Fig. 5a). In the common ancestor of *O. sativa* and *B. distachyon*, 2 ancestral genes were lost, and no ancestral gene was duplicated. Since the divergence, each of the *B. distachyon* and *O. sativa* lineages lost 3 genes, and there was no duplicated gene. In the common ancestor of *Z. mays*, *S. bicolor*, and *S. italica*, 3 ancestral genes were lost, and 2 ancestral genes were duplicated. In conclusion, there were more *BES1* gene losses than duplications. The ratio of losses and duplications was 28 versus 2 in dicots, and this ratio was 16 versus 3 in monocots. These results will help us to understand the loss, retention and expansion of this gene family during the evolution of different species.

### Exploring *BES1* gene function in *B. napus* by comparing it with *A. thaliana*

**Fig. 6:**
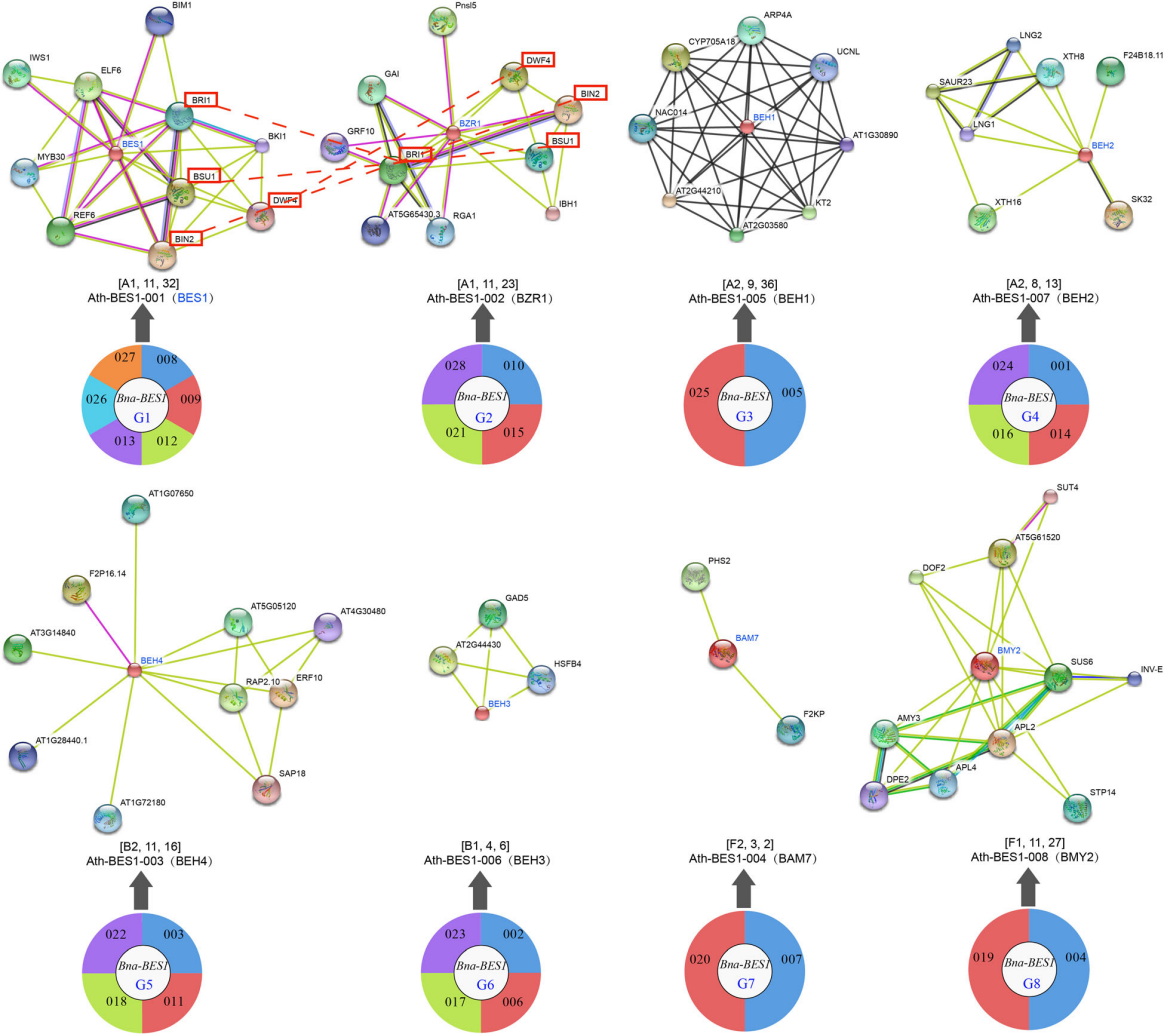
The construction of interaction networks for each *BES1* gene and their target genes in *A. thaliana*. The pie graph indicates the *B. napus BES1* genes involved in the related network of *A. thaliana*. The three numbers in the bracket indicate the groups, the gene number, and the connection edges

A comparison of sequence homologs between non-model and model species might aid in understanding the function of these *BES1* genes in non-model species. Taking 3 *Brassica* species as an example, we constructed three phylogenetic trees for these species and *A. thaliana*. On the phylogenetic tree, 8 groups (G1 to G8) were obtained according to the *BES1* genes of *A. thaliana* (Fig. 5b). There was no gene loss in G1, while there were 2 or 4 gene losses in the other 7 groups. The *BES1* genes within the same taxonomic group with *A. thaliana* might have similar functions. Therefore, we conducted comprehensive analyses to further explore the function of *BES1* genes. First, we constructed interaction networks between each *BES1* gene and their corresponding target genes to uncover their regulatory pathways (Fig. 6, Additional file 3: Tables S5, S6). Most target genes were identified in the networks of *Ath-BES1*, *Ath-ZR1*, *Ath-BEH4*, and *Ath-BMY2*, and the number of target genes achieved 10. Among all eight groups, there were the most *B. napus* genes involved in *Ath-BES1* networks. Interestingly, we identified 4 target genes (*BRI1*, *BSU1*, *BIN2*, and *DWF4*), which were involved in *BES1* and *BZR1* networks (Fig. 6). Furthermore, we conducted enrichment analyses for the genes involved in these networks, and found that most genes responded to the brassinosteroid signalling pathway (Additional file 3: Table S7).

### Comparative expression pattern analysis of *BES1* genes between *A. thaliana* and *B. napus*

**Fig. 7:**
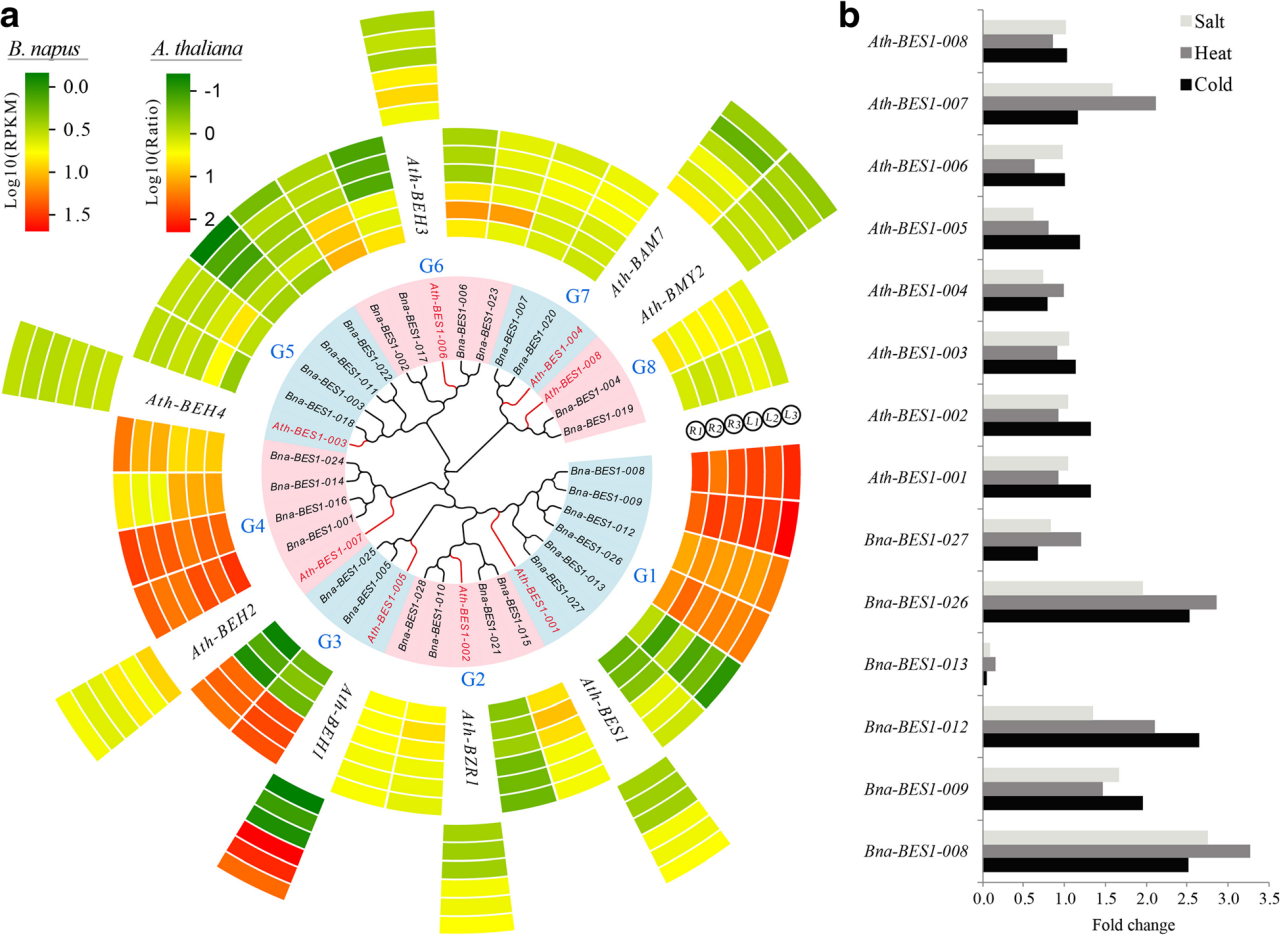
Expression pattern analysis of *BES1* genes in *A. thaliana* and *B. napus*. **a** Phylogenetic relationships were constructed and were divided into 8 groups (G1 to G8) according to the *A. thaliana BES1* genes. The genes of *A. thaliana*, subgenome A, and subgenome C of *B. napus* are marked with blue, green, and red, respectively. The gene expression in roots (R1, R2, and R3) and leaves (L1, L2, and L3) were determined by the AtGenExpress Visualization Tool and *B. napus* RNA-Seq. **b** The expression level of *BES1* genes in *B. napus* and *A. thaliana* under cold, heat, and salt treatments

**Fig. 8:**
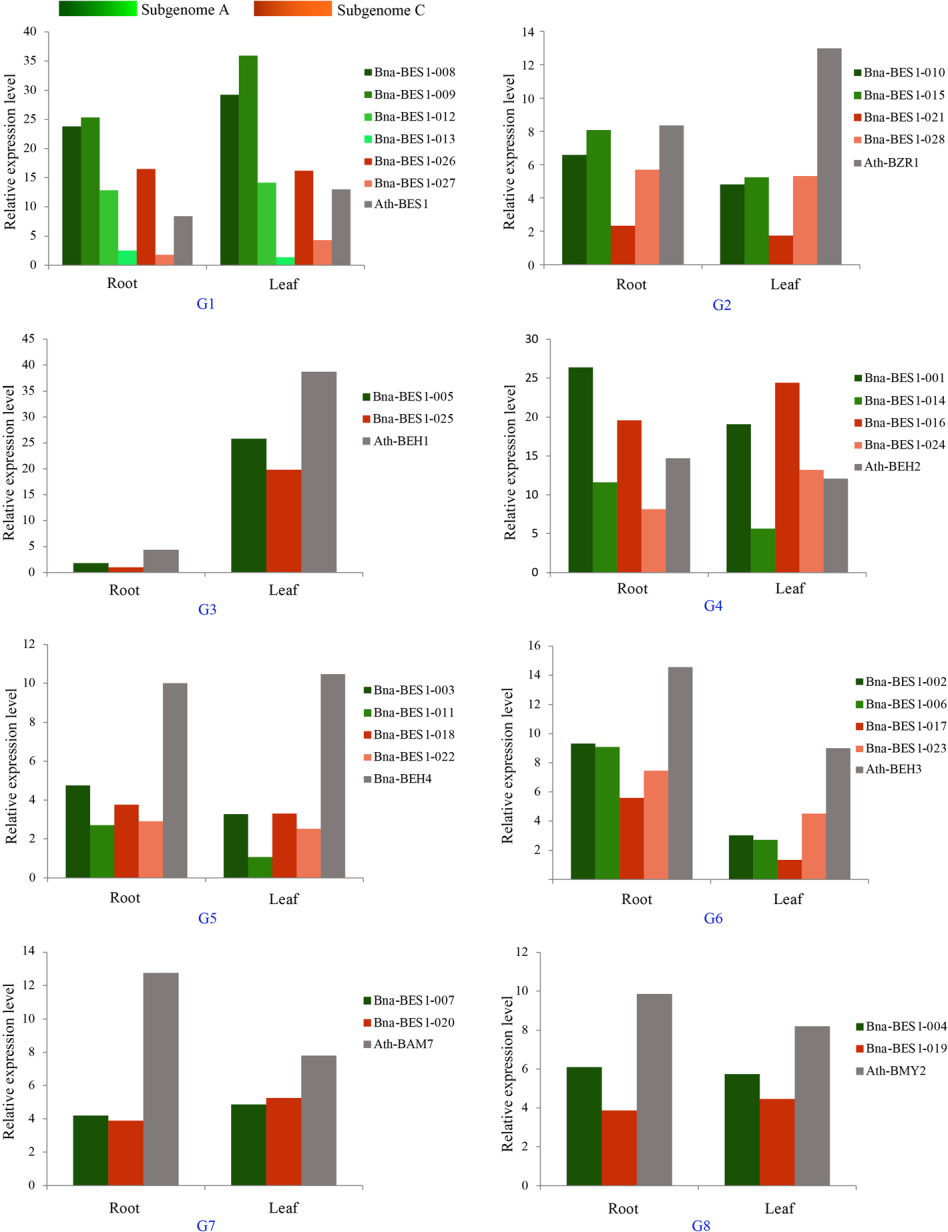
The histogram of *BES1* gene expression in roots and leaves of *B. napus* and *A. thaliana*. The G1 to G8 represent the 8 groups classified by *A. thaliana BES1* genes. The genes marked with green locate the subgenome A, and red locates the subgenome C of *B. napus*

To detect the functional divergence of *BES1* genes, their expression was compared in different tissues, i.e., roots and leaves, between *A. thaliana* (Additional file 3: Table S8) and *B. napus* (Fig. 7, Additional file 3: Table S9). The results showed that *Ath-BEH1* had high expression (log2 (ratio) > 1.5) in leaves, whereas it showed low expression in roots. Four genes (*Ath-BEH4*, *Ath-BEH3*, *Ath-BAM7*, *Ath-BMY2*) were expressed at low levels in leaves (log2 (ratio) < 0). In roots, 3 of 8 genes showed high expression, including *Ath-BAM7*, *Ath-BEH3*, and *Ath-BEH2*. For *B. napus*, we found that several genes showed high expression in both leaves and roots, such as all 4 genes in group 4 (G4) and four genes in G1 (*Bna-BES1–008,009,012, 026*). Interestingly, we found there was similar expression of these genes in the same group between *A. thaliana* and *B. napus*. For example, the expression of *Bna-BES1–005*, and *Bna-BES1–025* (G3) had similar patterns to *Ath-BEH1* in both roots and leaves. However, *Bna-BES1–028* and *Bna-BES1–010* were also located in G3, but their expression pattern was significantly different from *Ath-BEH1* (Fig. 7a). This might be due to the possibility of subfunctionalization or neofunctionalization after the divergence of *A. thaliana* and *B. napus*. In addition, we also found that the expression level of almost all genes in subgenome A was higher than that of subgenome C in roots (Fig. 8, Additional file 2: Figure S12).

Furthermore, we collected and analysed the expression of *BES1* genes in model species, which could be helpful for our understanding of homologous gene function in non-model species. Take the *BES1* gene as an example, we investigated its expression pattern in *A. thaliana* under different stresses, such as cold, heat, and salt (Fig. 7b). To explore the expression pattern of the duplicated genes, we conducted the qRT-PCR analyses for *BES1* genes in *B. napus* under cold, heat, and salt treatments. Among the G1 group, there were 6 duplicated genes in *B. napus* with the *A. thaliana BES1* gene. The results showed that *Bna-BES1–013* had a relatively low expression (Fig. 7b). This indicates that not all duplicated genes are highly expressed, and some genes may be de-functionalized after duplication.

## Discussion

### Origination, evolution and expansion of *BES1* family genes

The evolution and origin of *BES1* genes in representative plants were analysed, and their evolutionary pattern was determined using phylogenetic and conversed motif analyses. As to our phylogenetic analysis, *BES1* family genes may have first originated from the group F3, which contains only the M1 and M2 motifs. Then, it developed novel genes through acquiring other motifs, such as M2 to M10. Furthermore, several specific groups or subgroups were obtained for dicot and monocot species. In groups F1 and F2, we detected an additional domain contained M4 to M8, which did not exist in the other groups. The WGD or segmental duplication contributed the most to the expansion of this gene family among all 6 dicots [47, 48]. However, the dispersed duplication contributed the most to its expansion in 3 monocots. The percentage of *BES1* genes located in collinear blocks was significant larger than the average genome-wide level for nearly all examined species.

The *Brassica* genome has undergone genome duplication events since its divergence from *A. thaliana* [47, 49–51]. Furthermore, *B. napus*, an allopolyploid, originated by hybridization between *B. rapa* and *B. oleracea* only ~ 7500 years ago [19]. Therefore, the *A. thaliana* gene should have 3 corresponding *B. oleracea* and *B. rapa* genes and have 6 *B. napus* genes if no gene was lost. However, there is a high possibility of gene loss after WGD events. For all 7 *A. thaliana BES1* genes except *Ath-BES1*, the loss events occurred in *B. rapa* and *B. oleracea* (Fig. 5b). On inspection, *B. napus BES1* genes were lost when comparing with *A. thaliana* genes. In fact, we thought the *BES1* gene was not lost, because *B. napus* originated by hybridization of *B. rapa* and *B. oleracea*, and the loss occurred during the fusion of these two genomes.

### *BES1* gene expression pattern and network construction in *B. napus* and model species

The BR signalling pathway has been studied thoroughly at the genetic and molecular level in *A. thaliana* [52–54]. The studies showed that *BZR1* and *BES1* are important components of BR signal transduction [55, 56]. Plants with impaired BR production exhibit many growth defects, such as dwarfism, or organ expansion reduction [57, 58]. *BES1* genes repress the expression of several BR biosynthesis enzymes, such as *CPD* and *DWARF4* [59, 60]. Based on the microarray and ChIP-chip data, 1609 and 953 BR-regulated target genes have been identified for *BES1* and *BZR1*, respectively [15, 61]. The construction of an expression detection or interaction network will aid in revealing the regulation of *BES1* genes.

In this study, we conducted expression analyses of *BES1* genes in *B. napus* and *A. thaliana* by using RNA-Seq and qRT-PCR datasets. In *B. napus*, most genes of the G1 and G4 groups showed higher expression in both roots and leaves. Interestingly, we found that not all duplicated genes were highly expressed. In the G1 group, there were 4 and 2 homologous genes with the *A. thaliana BES1* gene in subgenome A and C, respectively. However, the *Bna-BES1–013* (from subgenome A) and *Bna-BES1–027* (from subgenome C) had a lower expression level than the other 4 genes in roots and leaves. Similarly, this phenomenon was also found under cold, heat, and salt treatments. The results indicated that some genes may be sub-, neo- or defunctionalized during the species’ evolution. Furthermore, we conducted interaction networks for *B. napus* and *A. thaliana*. The network was more complex for *BES1*, *BZR1*, and *BEH1* than for other genes. This indicated that they may play a more important core role in the regulation network. Comparative analysis of the expression pattern and construction of the regulatory network will provide a very favourable support for the study of *BES1* gene function in *B. napus* and other related species.

### Exploring *BES1* gene function in non-model species

The functions of most *BES1* genes have been well characterized in *A. thaliana*. *BES1* targets are not only auxin responsive genes, but are also auxin efflux facilitators (*PIN4, PIN7*), which have profound effects on auxin action [15, 62]. Based on microarray and chromatin immune precipitation (ChIP), *AtMYB30* was identified, which is a direct target gene of *AtBES1* [63]. By spectrometry identification and genetic analysis, it was found that *MAX2* mediates the ubiquitination and degradation of BES1 protein [64]. In addition, the *BES1* gene is a direct substrate for *MPK6* to regulate the immune response of plants [65]. *BZR1* mediates a trade-off between plant innate immunity and growth [66]. Therefore, a comparison of sequence homologs between model and non-model species might aid in understanding the function of these *BES1* genes in non-model species. Taking the Brassicaceae species as an example, we checked genes within the same taxonomic group on the phylogenetic tree, which could have similar functions (Fig. 5b).

We identified 3 *BES1* genes (*Bra-BES1–010, Bra-BES1–013, Bra-BES1–014*) in *B. rapa*, 3 *BES1* genes (*Bol-BES1–006, Bol-BES1–012, Bol-BES1–013*) in *B. oleracea*, and 6 *BES1* genes in *B. napus*, which clustered together with the *BES1* gene of *A. thaliana* (Fig. 5b), functionally related to cooperating with transcription factors to regulate BR-induced genes [2, 3]. Similarly, several homologous genes in three Brassica species were clustered together with other *BES1* family genes, such as *BZR1*, *BEH1–4* (Fig. 5b), functionally involving BRs regulation [13, 55, 64–66]. These comprehensive and comparative analyses in *B. napus* and model species provides rich valuable resources for understanding the *BES1* genes’ function and regulatory mechanisms in *B. napus* and other non-model species.

## Conclusions

In conclusion, we comprehensively analysed the evolutionary pattern, gene synteny, gene duplication or losses, orthologous and paralogous genes, positive selection, and interaction networks of *BES1* genes involved in the BRs pathway. A total of 132 *BES1* genes were identified among 19 representative species. This effort can serve as a first step in comprehensive functional characterization of *BES1* genes by reverse genetic approaches and molecular genetics research. This study provides useful resources for future studies on the structure and function of *BES1* and for identifying and characterizing these genes in other species. In addition, this study may also facilitate our understanding of the effect of duplication or losses during the evolution of polyploidy.

## Additional files

Additional file 1: **Figure S1.** The positive selection analyses for each group of BES1 gene family in representative species. The ω on the clades is dn/ds value under M8 model of codeml, which indicates the positive selection nodes. (PDF 634 kb)

Additional file 2: **Figure S2.** The sequences of motif 1 to motif 10 of BES1 gene family among examined species. Figure S3. The conversed motifs (M1 to M10) analyses for each group using the MEME program. Figure S4. The sequences alignment of F1, F2, and F3 subgroups among examined species. The Glyco_hydro_14 domain contained in F1 and F2 subgroups is marked using the blue dashed box in F3 subgroup. Figure S5. The analyses of Glyco_hydro_14 domain, including (a) protein structure, (b) alignment with the homology model (1fa2.1.A), (c) prediction of local similarity to target, (d) comparison with non-redundant set of PDB structures. Figure S6. The analyses of orthologous *BES1* genes between *O. sativa* and other examined species. (a) The interaction network constructed using the orthologous gene pairs between *O. sativa* and other species. (b) The number of orthologous genes in each species with 6 *O. sativa BES1* genes. Figure S7. The analyses of orthologous *BES1* genes between *A. trichopoda* and other examined species. (a) The interaction network constructed using the orthologous gene pairs between *A. trichopoda* and other species. (b) The number of orthologous genes in each species with 4 *A. trichopoda BES1* genes. Figure S8. The analyses of orthologous *BES1* genes between *S. moellendorffii* and other examined species. (a) The interaction network constructed using the orthologous gene pairs between *S. moellendorffii* and other species. (b) The number of orthologous genes in each species with 4 *S. moellendorffii BES1* genes. Figure S9. The analyses of orthologous *BES1* genes between *P. patens* and other examined species. (a) The interaction network constructed using the orthologous gene pairs between *P. patens* and other species. (b) The number of orthologous genes in each species with 6 *P. patens BES1* genes. Figure S10. The analyses of paralogous *BES1* genes in each of the examined species. The green lines indicated the 4 *B. napus BES1* genes, which had more than 1 paralogous genes. Figure S11. The reconstructed phylogenetic tree according to the duplication and losses of *BES1* genes at different stages of plant evolution. Figure S12. The histogram of *BES1* genes expression in root and leaf of *B. napus.* The genes marked with green located subgenome A, and red located subgenome C of *B. napus*. (PDF 4242 kb)

Additional file 3: **Table S1.** The list of orthologous *BES1* gene pairs between *B. napus*, *A. thaliana*, *O. sativa*, *A. trichopoda*, *S. moellendorffii*, *P. patens* and other examined species. Table S2. The list of paralogous *BES1* gene pairs in each of other examined species. Table S3. The duplicated type of *BES1* genes in *B. napus* and each representative species. Table S4. The comparative of synteny analyses for *BES1* genes and all genes in *B. napus* and other representative species. Table S5. The domain and annotation of the *BES1* genes and their target genes in *A. thaliana* network for comparative ananlysis with *B. napus*. Table S6. The nodes information and the assessment of the interaction network for *BES1* genes in *A. thaliana*. Table S7. The enrichment ananlyses of the genes involved in the *BES1* genes networks in *A. thaliana* for comparative ananlysis with *B. napus.* Table S8. The expression information of the *BES1* genes in root and leaf for *A. thaliana*, and the gene expression was obtained from the BAR database. Table S9. The expression level of the *BES1* genes in root and leaf for *B. napus* and *A. thaliana*. The gene expression was determined by the *B. napus* RNA-Seq data (RPKM) and the BAR database for *A. thaliana*. (XLS 159 kb)

## Abbreviations

*BES1*: BRI1-EMS-SUPPRESSOR1
BIM1: *BES1*-interacting Myc-like1
BRs: Brassinosteroids
ChIP: Chromatin immune precipitation
JTT model: Jones, Taylor and Thorton model
LR tests: *L*ikelihood ratio tests
NLS: Nuclear localization signal
WGD: Whole-genome duplication
