# Supplementary material for: Comprehensive analyses of the BES1 gene family in Brassica napus and examination of their evolutionary pattern in representative species
Source: BMC Genomics. 2018 May 9;19:346. doi: 10.1186/s12864-018-4744-4 (PMC5944053; doi:10.1186/s12864-018-4744-4)
Supplement: Supplementary file 1 — Figure S1. The positive selection analyses for each group of BES1 gene family in representative species. The ω on the clades is dn/ds value under M8 model of codeml, which indicates the positive selection nodes. (PDF 634 kb) [file 12864_2018_4744_MOESM1_ESM.pdf]

Large-scale analyses of BES1 gene family reveal the distinct evolutionary patterns between *Brassicaceae* and *Poaceae* species

Xiaoming Song[1], Xiao Ma[2], Chunjin Li[1], Jingjing Hu[1], Qihang Yang[1], Tong Wang[1], Li Wang[1], Jinpeng Wang[1], Di Guo[1], Weina Ge[1], Zhenyi Wang[1], Lan Zhang[1], Jiaqi Zhang[1], Xiyin Wang[1,*]

[1]Center of Genomics and Computational Biology, College of Life Sciences, North China University of Science and Technology, Tangshan, Hebei 063000, China.

[2]Library, North China University of Science and Technology, Tangshan, Hebei 063000, China.

***Corresponding author:** Xiyin Wang

E-mail: wang.xiyin@gmail.com

Figure S1. The positive selection analyses for each group of BES1 gene family in representative species. The ω on the clades is dn/ds value under M8 model of codeml, which indicates the positive selection nodes.

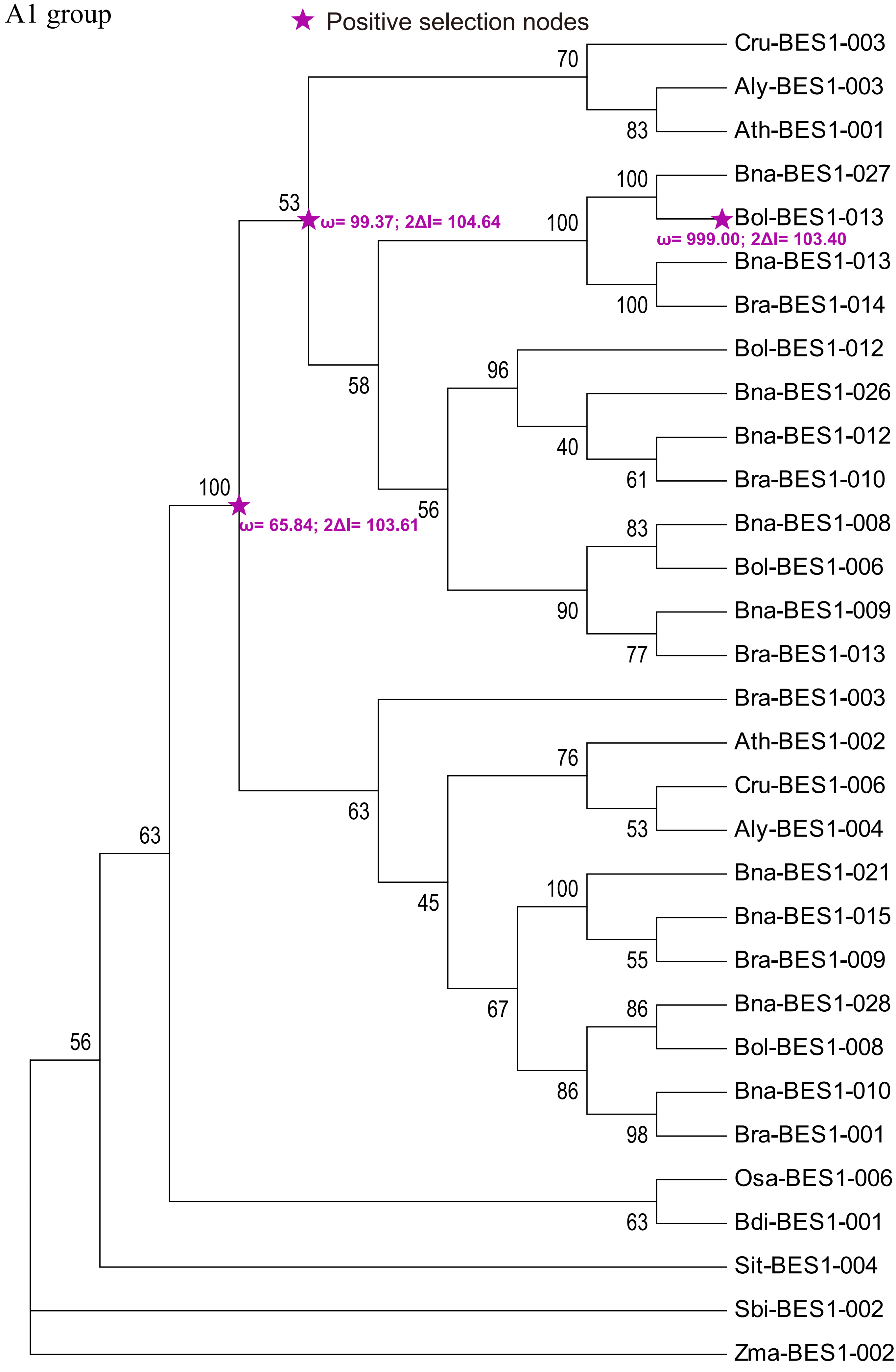
A1 group
Positive selection nodes
ω= 99.37; 2Δl= 104.64
ω= 999.00; 2Δl= 103.40
ω= 65.84; 2Δl= 103.61
Cru-BES1-003
Aly-BES1-003
Ath-BES1-001
Bna-BES1-027
Bol-BES1-013
Bna-BES1-013
Bra-BES1-014
Bol-BES1-012
Bna-BES1-026
Bna-BES1-012
Bra-BES1-010
Bna-BES1-008
Bol-BES1-006
Bna-BES1-009
Bra-BES1-013
Bra-BES1-003
Ath-BES1-002
Cru-BES1-006
Aly-BES1-004
Bna-BES1-021
Bna-BES1-015
Bra-BES1-009
Bna-BES1-028
Bol-BES1-008
Bna-BES1-010
Bra-BES1-001
Osa-BES1-006
Bdi-BES1-001
Sit-BES1-004
Sbi-BES1-002
Zma-BES1-002

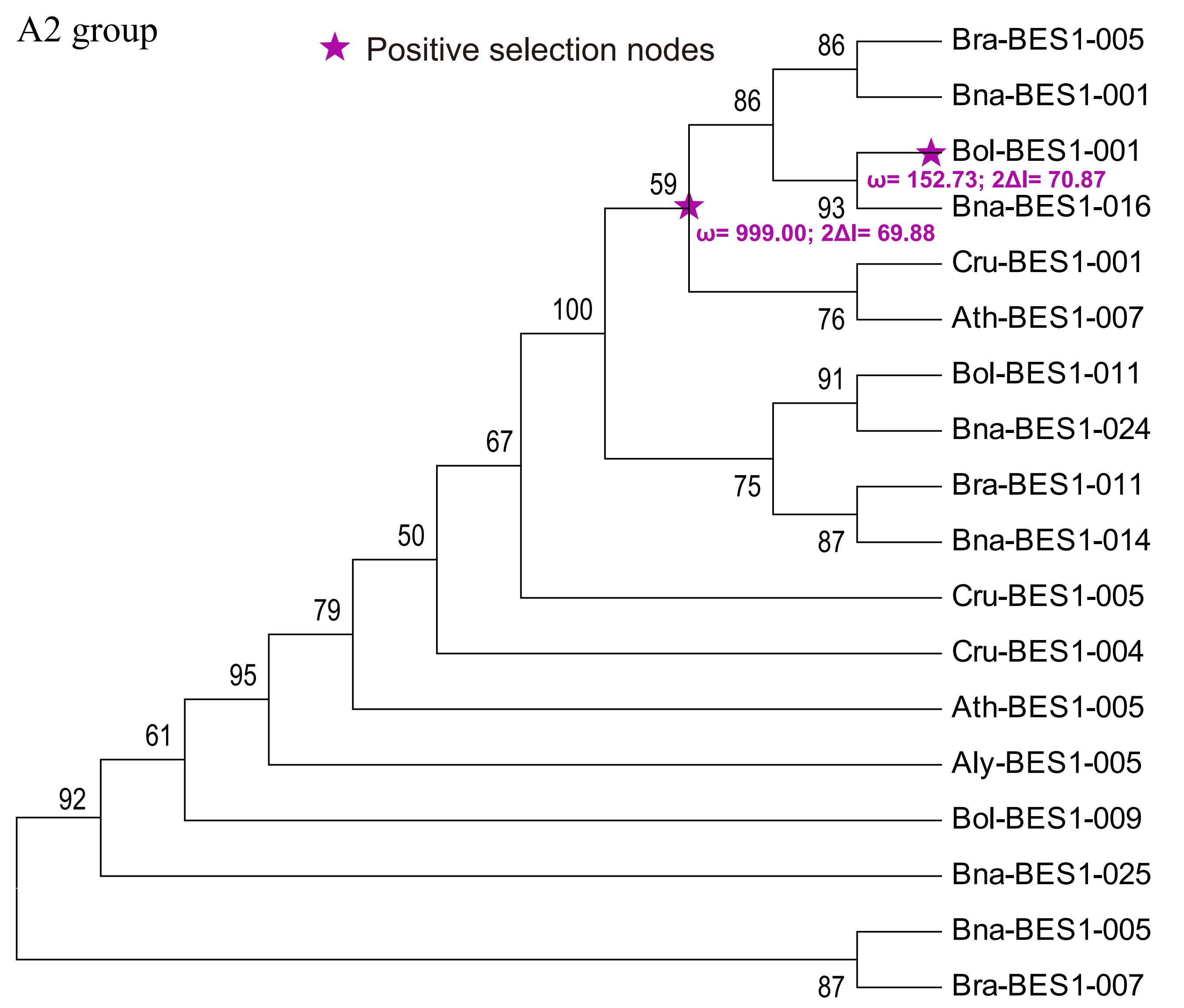
A2 group
Positive selection nodes
Bra-BES1-005
Bna-BES1-001
Bol-BES1-001
ω= 152.73; 2Δl= 70.87
Bna-BES1-016
ω= 999.00; 2Δl= 69.88
Cru-BES1-001
Ath-BES1-007
Bol-BES1-011
Bna-BES1-024
Bra-BES1-011
Bna-BES1-014
Cru-BES1-005
Cru-BES1-004
Ath-BES1-005
Aly-BES1-005
Bol-BES1-009
Bna-BES1-025
Bna-BES1-005
Bra-BES1-007
86
86
93
59
76
100
91
75
87
67
50
79
95
61
92
87

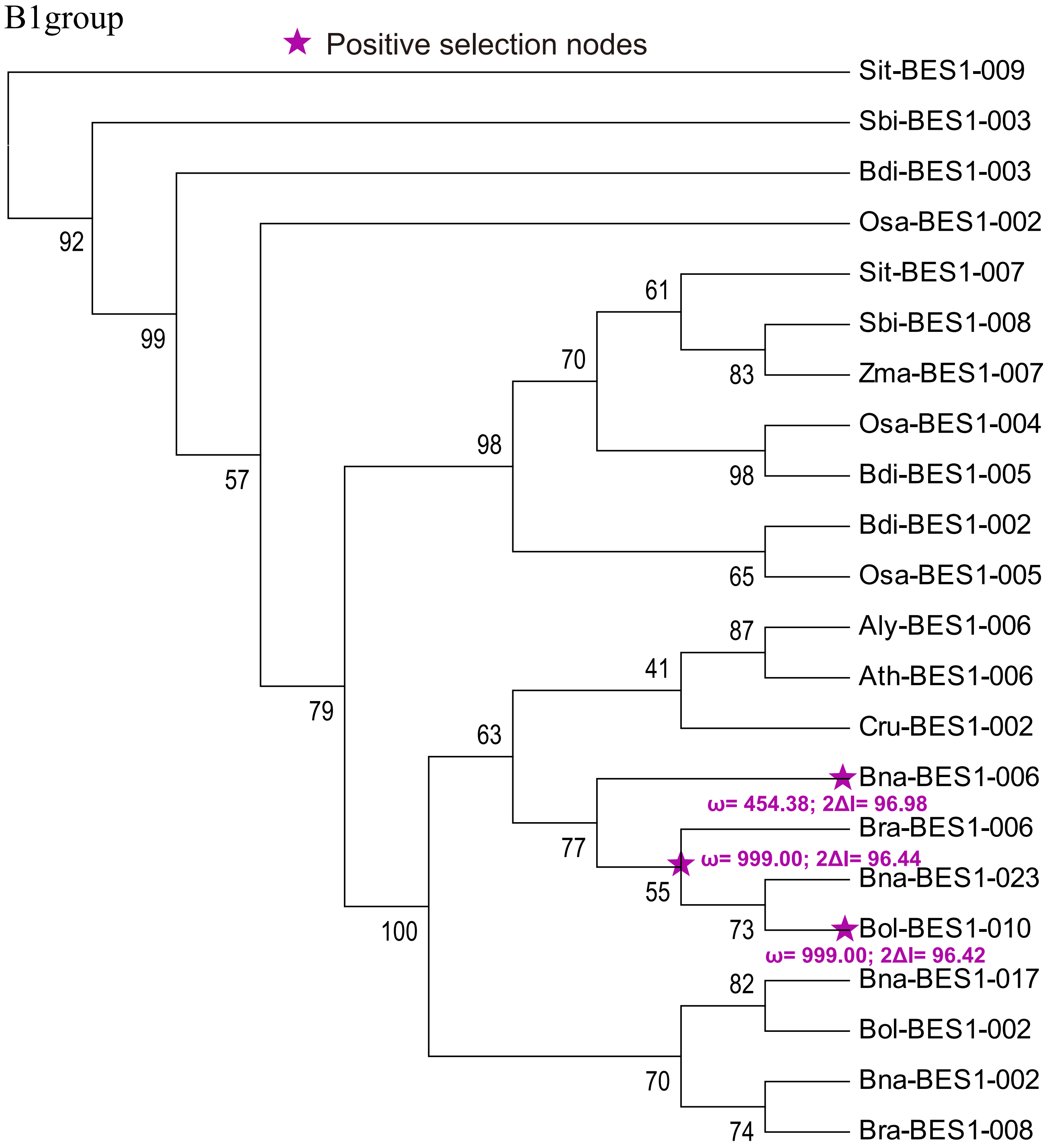
B1group
Positive selection nodes
Sit-BES1-009
Sbi-BES1-003
Bdi-BES1-003
Osa-BES1-002
Sit-BES1-007
Sbi-BES1-008
Zma-BES1-007
Osa-BES1-004
Bdi-BES1-005
Bdi-BES1-002
Osa-BES1-005
Aly-BES1-006
Ath-BES1-006
Cru-BES1-002
Bna-BES1-006
ω= 454.38; 2Δl= 96.98
Bra-BES1-006
ω= 999.00; 2Δl= 96.44
Bna-BES1-023
Bol-BES1-010
ω= 999.00; 2Δl= 96.42
Bna-BES1-017
Bol-BES1-002
Bna-BES1-002
Bra-BES1-008
92
99
57
79
100
98
70
61
83
98
65
87
41
63
77
55
73
82
70
74

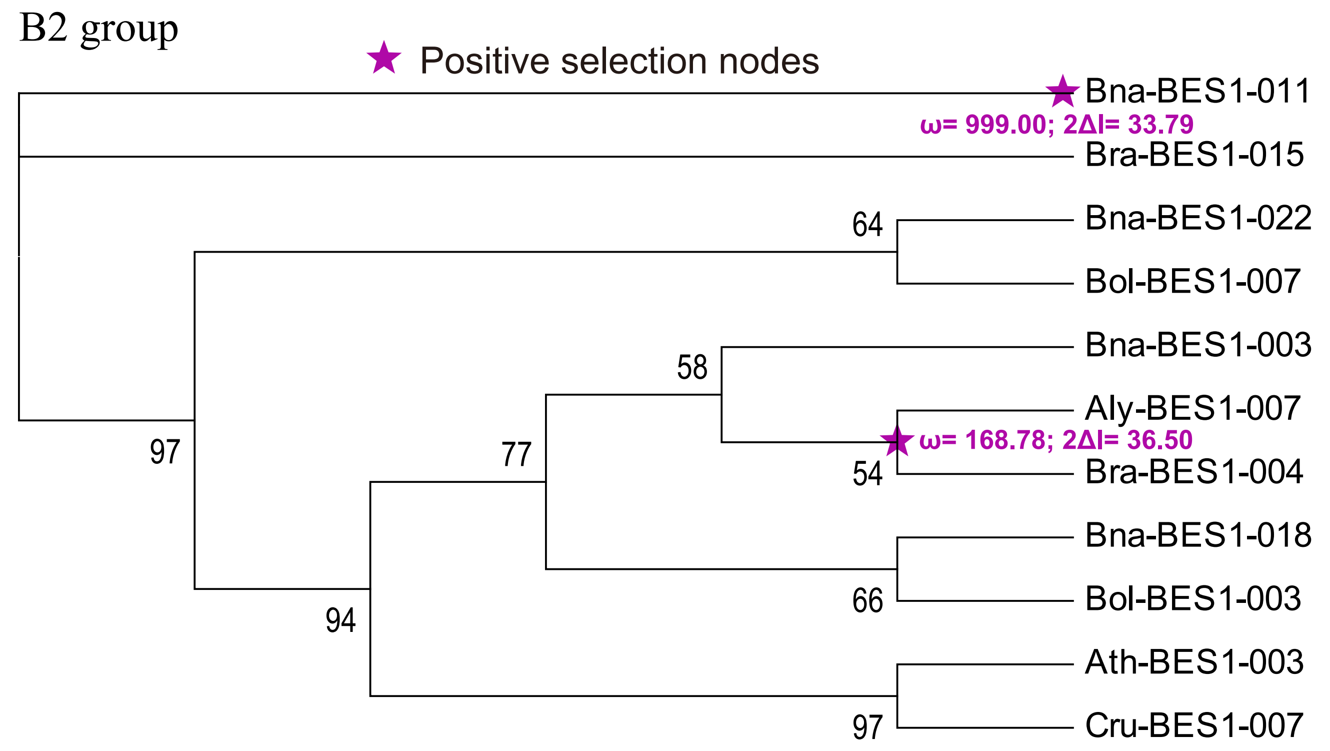
B2 group
Positive selection nodes
Bna-BES1-011
ω= 999.00; 2Δl= 33.79
Bra-BES1-015
64
Bna-BES1-022
Bol-BES1-007
58
Bna-BES1-003
Aly-BES1-007
ω= 168.78; 2Δl= 36.50
54
Bra-BES1-004
97
77
Bna-BES1-018
66
Bol-BES1-003
94
Ath-BES1-003
97
Cru-BES1-007

D group

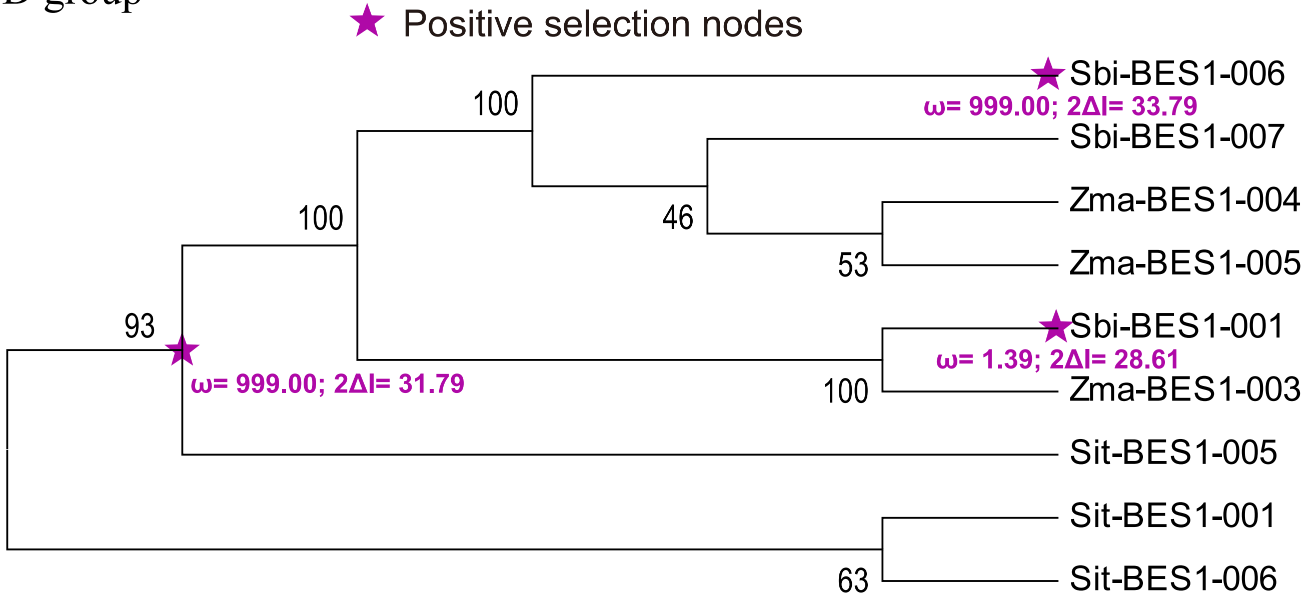

Positive selection nodes
Sbi-BES1-006
ω= 999.00; 2Δl= 33.79
Sbi-BES1-007
Zma-BES1-004
Zma-BES1-005
Sbi-BES1-001
ω= 1.39; 2Δl= 28.61
Zma-BES1-003
Sit-BES1-005
Sit-BES1-001
Sit-BES1-006
100
100
46
53
93
ω= 999.00; 2Δl= 31.79
100
63

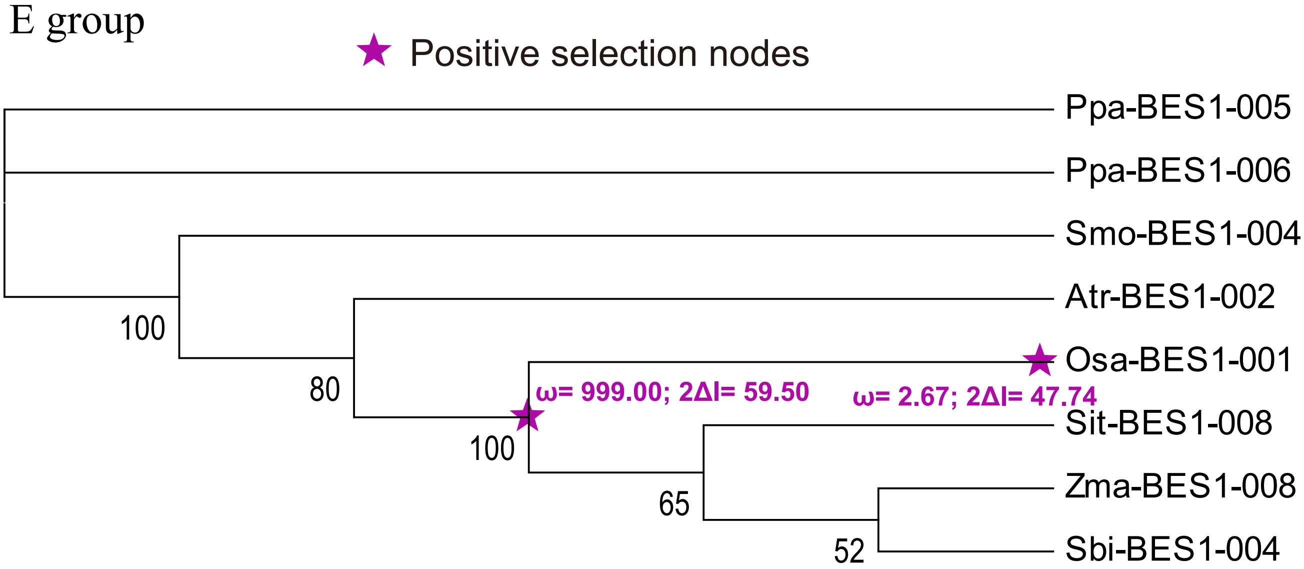
E group
Positive selection nodes
Ppa-BES1-005
Ppa-BES1-006
Smo-BES1-004
Atr-BES1-002
Osa-BES1-001
Sit-BES1-008
Zma-BES1-008
Sbi-BES1-004
100
80
100
65
52
ω= 999.00; 2Δl= 59.50
ω= 2.67; 2Δl= 47.74

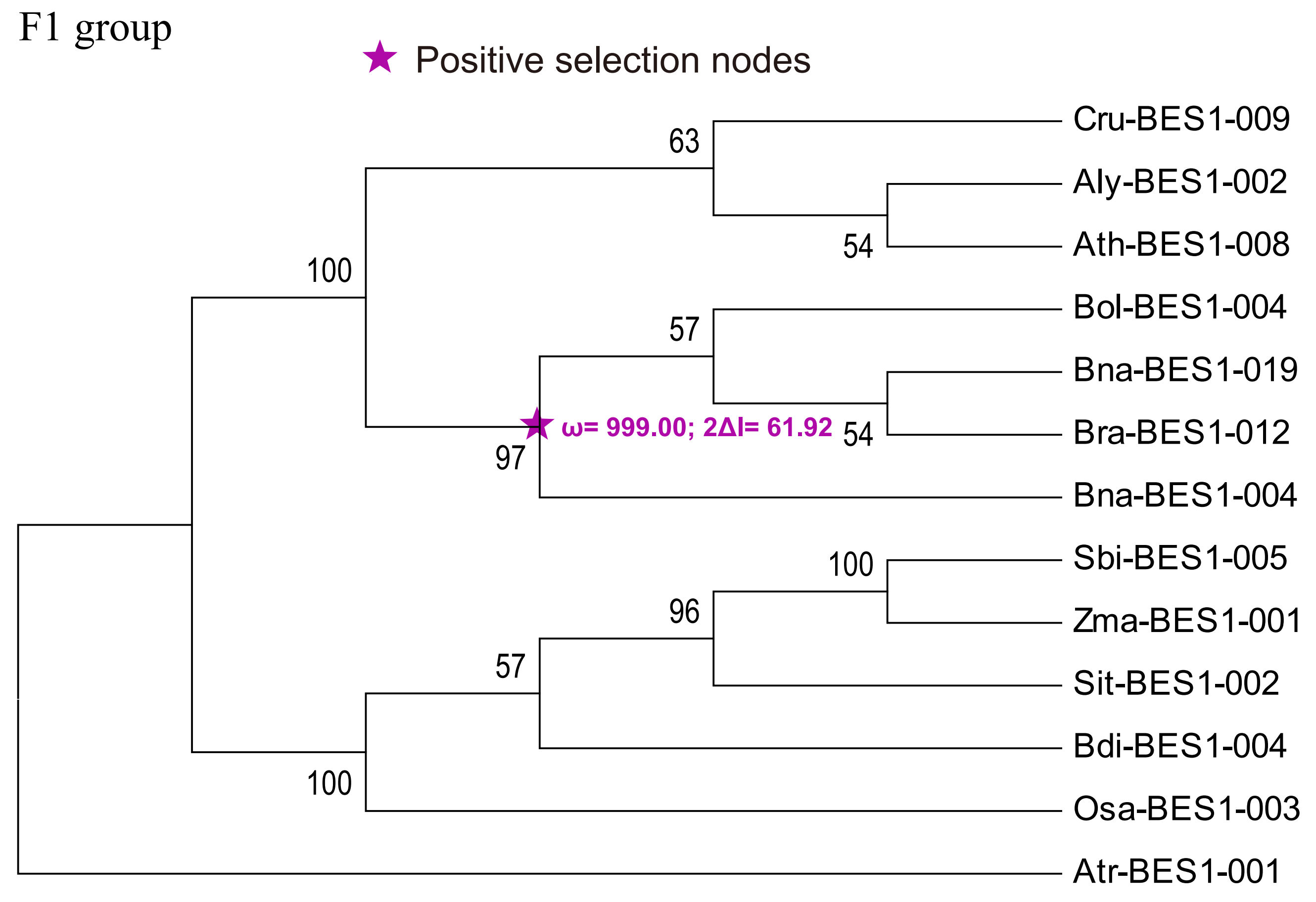
F1 group
Positive selection nodes
ω= 999.00; 2Δl= 61.92
63
54
100
57
54
97
100
96
57
100
Cru-BES1-009
Aly-BES1-002
Ath-BES1-008
Bol-BES1-004
Bna-BES1-019
Bra-BES1-012
Bna-BES1-004
Sbi-BES1-005
Zma-BES1-001
Sit-BES1-002
Bdi-BES1-004
Osa-BES1-003
Atr-BES1-001

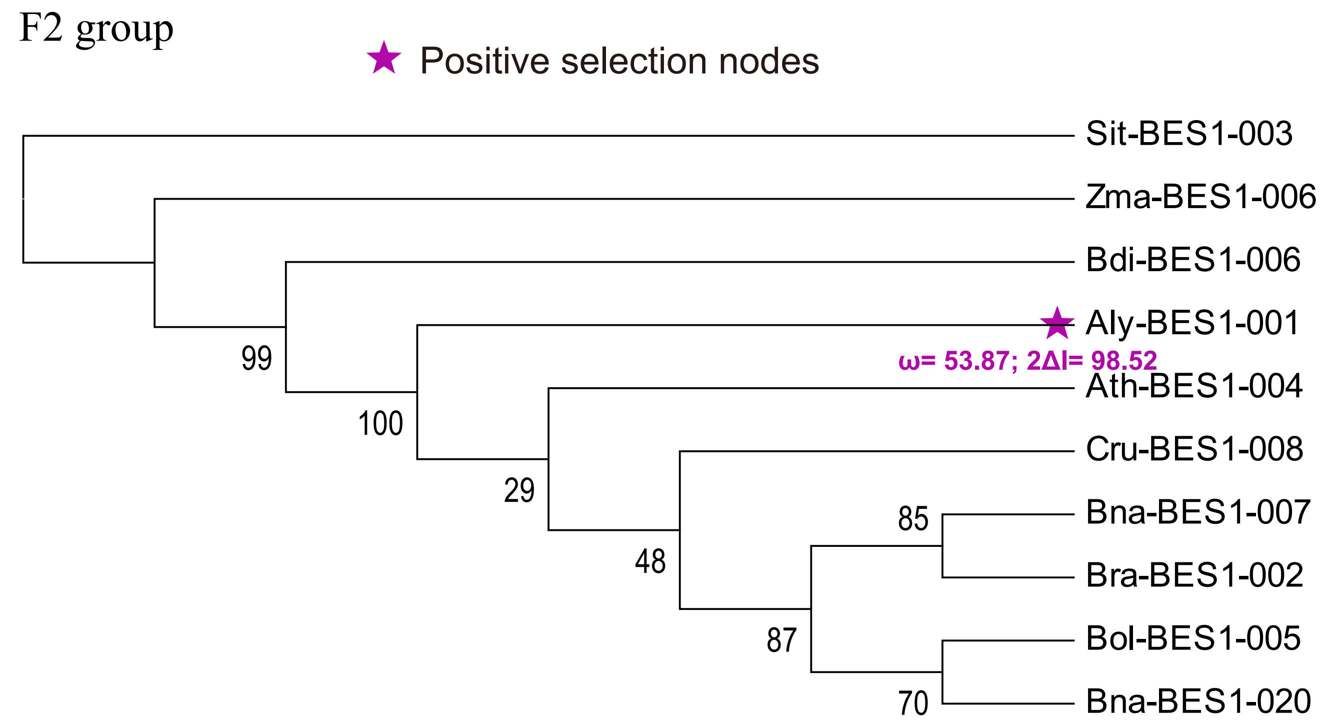
F2 group
Positive selection nodes
Sit-BES1-003
Zma-BES1-006
Bdi-BES1-006
Aly-BES1-001
ω= 53.87; 2Δl= 98.52
Ath-BES1-004
Cru-BES1-008
Bna-BES1-007
Bra-BES1-002
Bol-BES1-005
Bna-BES1-020
99
100
29
48
87
85
70

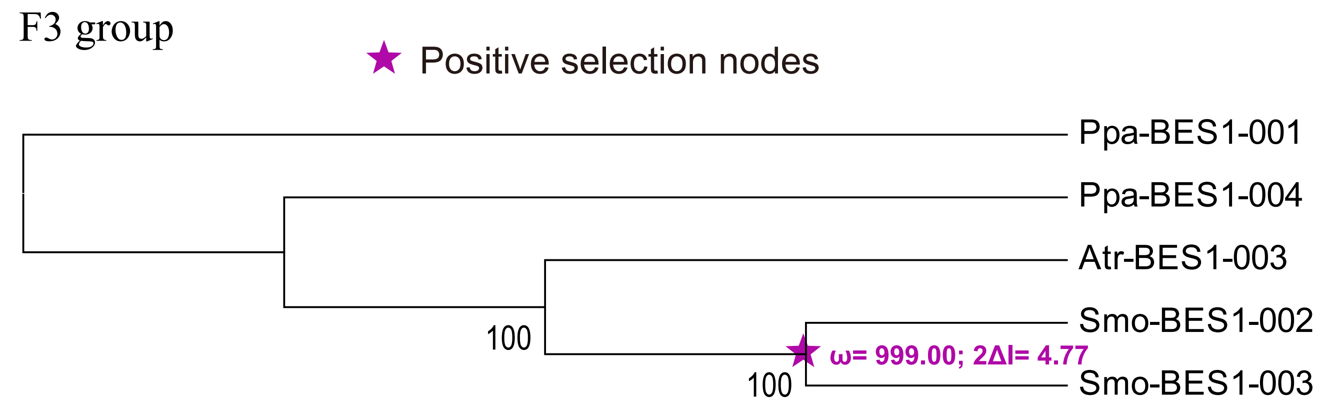
F3 group
Positive selection nodes
Ppa-BES1-001
Ppa-BES1-004
Atr-BES1-003
Smo-BES1-002
Smo-BES1-003
100
100
ω= 999.00; 2Δl= 4.77
