# Supplementary material for: Comprehensive analyses of the BES1 gene family in Brassica napus and examination of their evolutionary pattern in representative species
Source: BMC Genomics. 2018 May 9;19:346. doi: 10.1186/s12864-018-4744-4 (PMC5944053; doi:10.1186/s12864-018-4744-4)
Supplement: Supplementary file 2 — Figure S2. The sequences of motif 1 to motif 10 of BES1 gene family among examined species. Figure S3. The conversed motifs (M1 to M10) analyses for each group using the MEME program. Figure S4. The sequences alignment of F1, F2, and F3 subgroups among examined species. The Glyco_hydro_14 domain contained in F1 and F2 subgroups is marked using the blue dashed box in F3 subgroup. Figure S5. The analyses of Glyco_hydro_14 domain, including (a) protein structure, (b) alignment with the homology model (1fa2.1.A), (c) prediction of local similarity to target, (d) comparison with non-redundant set of PDB structures. Figure S6. The analyses of orthologous BES1 genes between O. sativa and other examined species. (a) The interaction network constructed using the orthologous gene pairs between O. sativa and other species. (b) The number of orthologous genes in each species with 6 O. sativa BES1 genes. Figure S7. The analyses of orthologous BES1 genes between A. trichopoda and other examined species. (a) The interaction network constructed using the orthologous gene pairs between A. trichopoda and other species. (b) The number of orthologous genes in each species with 4 A. trichopoda BES1 genes. Figure S8. The analyses of orthologous BES1 genes between S. moellendorffii and other examined species. (a) The interaction network constructed using the orthologous gene pairs between S. moellendorffii and other species. (b) The number of orthologous genes in each species with 4 S. moellendorffii BES1 genes. Figure S9. The analyses of orthologous BES1 genes between P. patens and other examined species. (a) The interaction network constructed using the orthologous gene pairs between P. patens and other species. (b) The number of orthologous genes in each species with 6 P. patens BES1 genes. Figure S10. The analyses of paralogous BES1 genes in each of the examined species. The green lines indicated the 4 B. napus BES1 genes, which had more than 1 paralogous genes. Figure S11. The recons [file 12864_2018_4744_MOESM2_ESM.pdf]

Supplementary Figure S2-S15

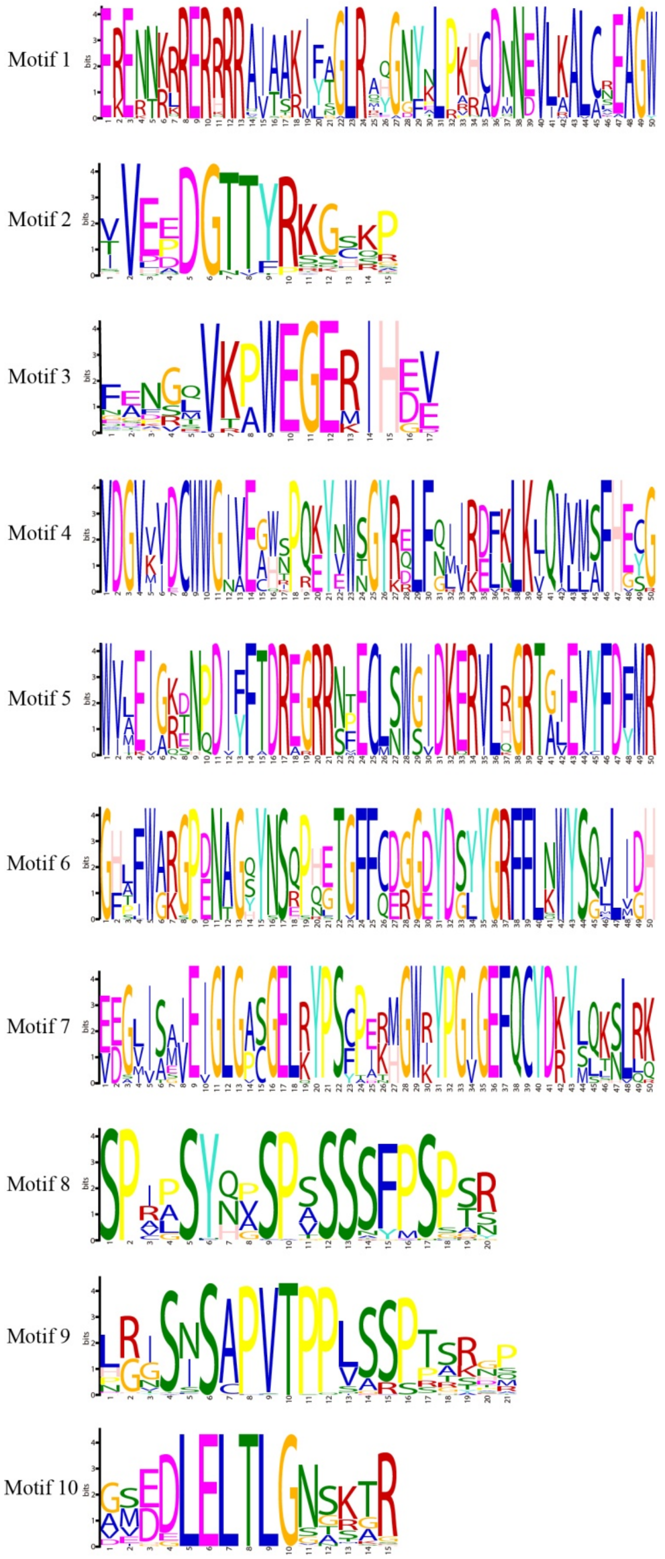

Figure S2. The sequences of motif 1 to motif 10 of BES1 gene family among examined species.

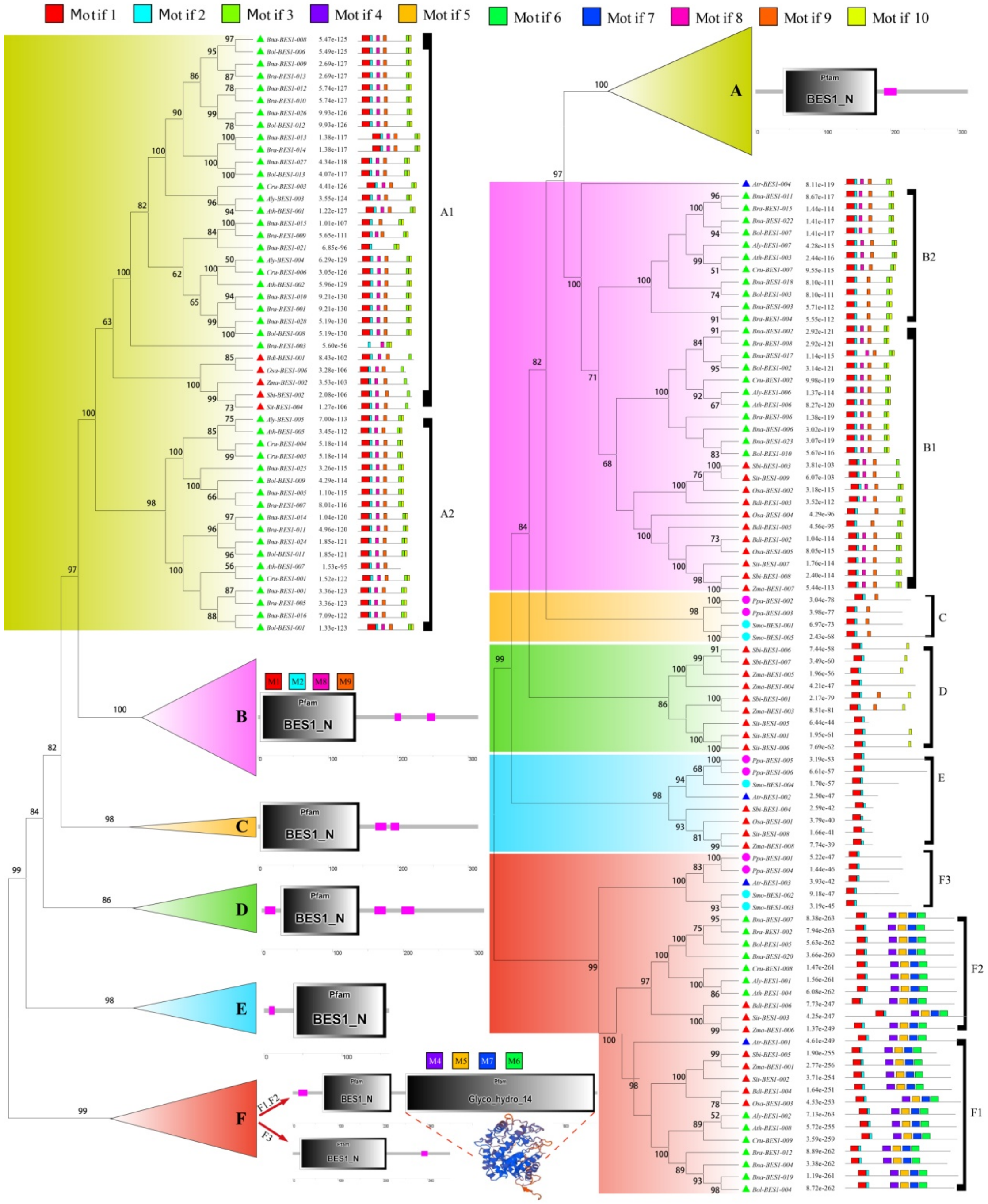

Figure S3. The conversed motifs (M1 to M10) analyses for each group using the MEME program.

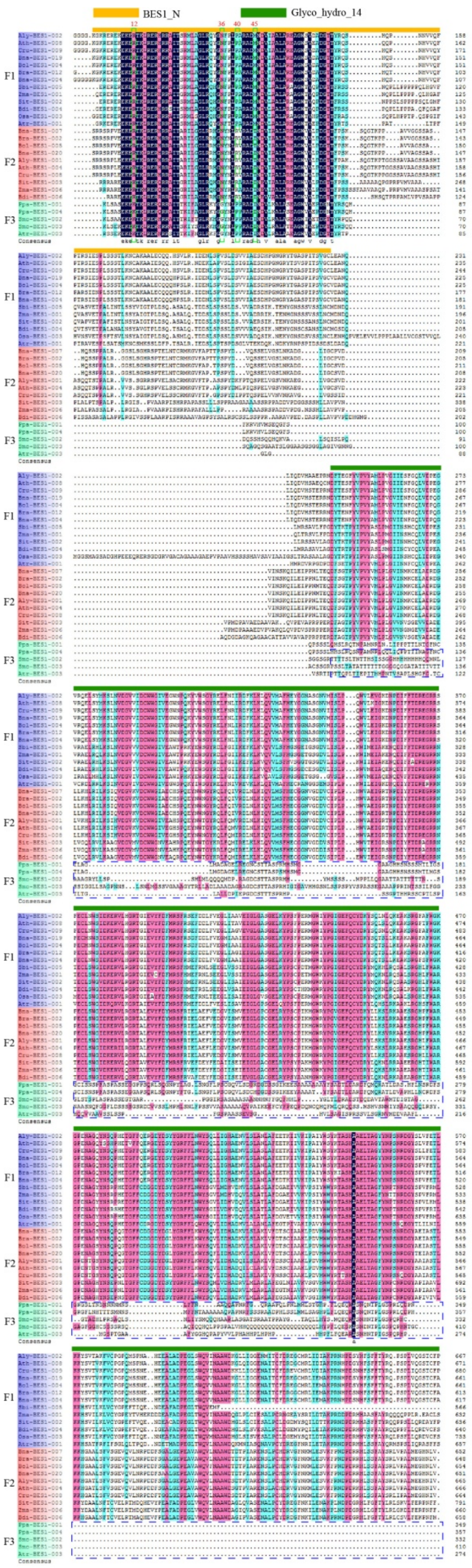

Figure S4. The sequences alignment of F1, F2, and F3 subgroups among examined species. The Glyco_hydro_14 domain contained in F1 and F2 subgroups is marked using the blue dashed box in F3 subgroup.

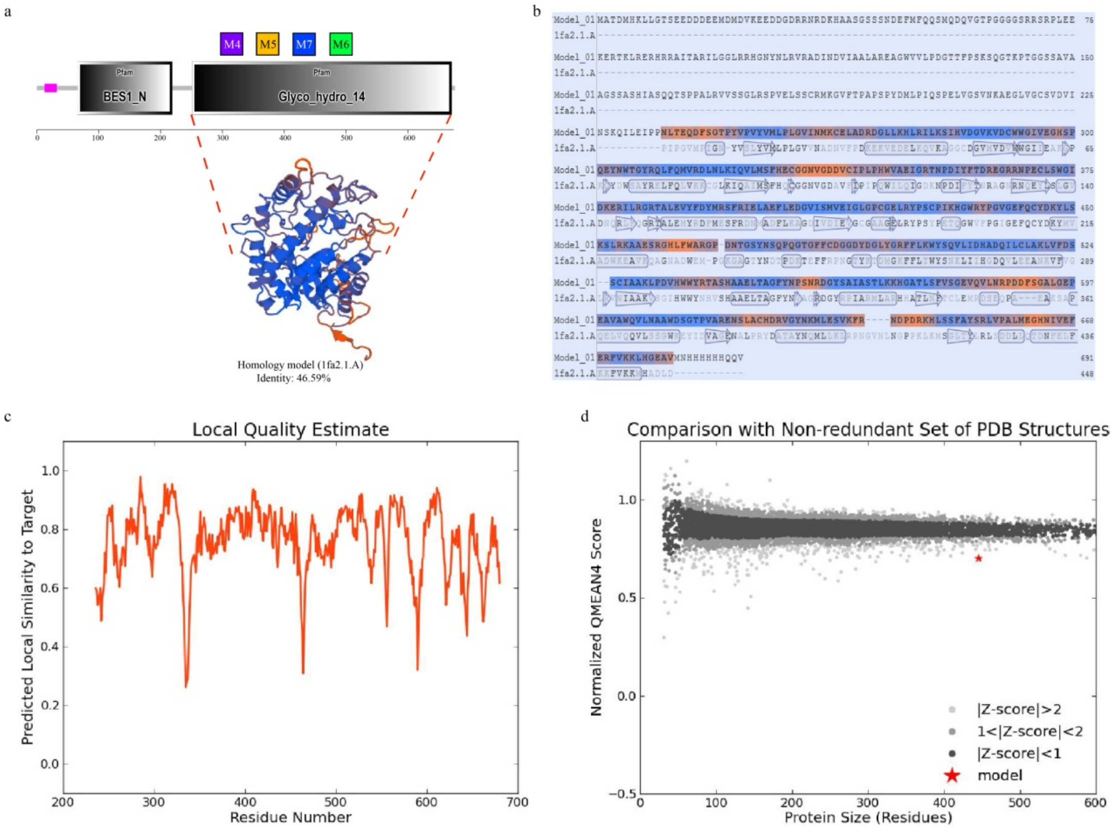

Figure S5. The analyses of Glyco_hydro_14 domain, including (a) protein structure, **(b)** alignment with the homology model (1fa2.1.A), **(c)** prediction of local similarity to target, **(d)** comparison with non-redundant set of PDB structures.

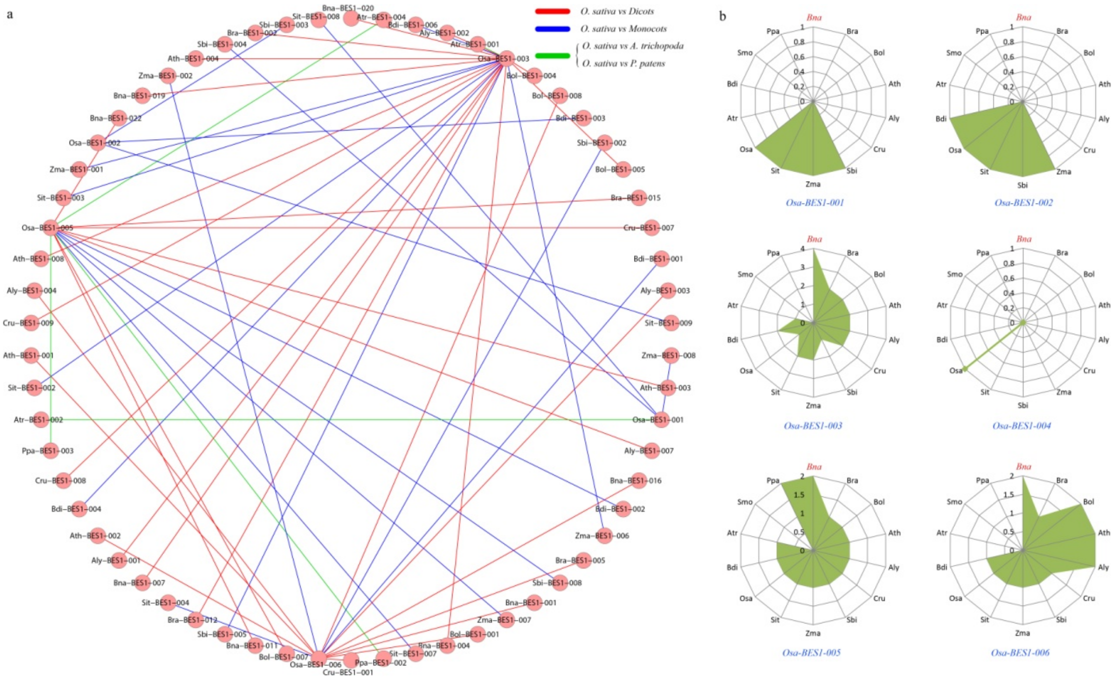

Figure S6. The analyses of orthologous *BES1* genes between *O. sativa* and other examined species. (a) The interaction network constructed using the orthologous gene pairs between *O. sativa* and other species. **(b)** The number of orthologous genes in each species with 6 *O. sativa BES1* genes.

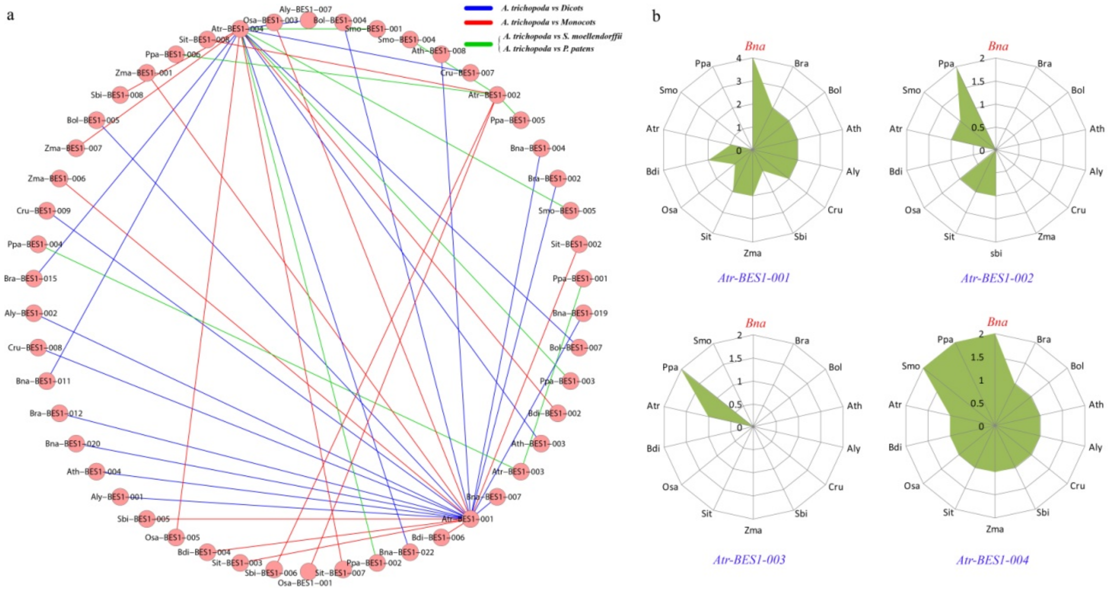

Figure S7. The analyses of orthologous *BES1* genes between *A. trichopoda* and other examined species. (a) The interaction network constructed using the orthologous gene pairs between *A. trichopoda* and other species. **(b)** The number of orthologous genes in each species with 4 *A. trichopoda BES1* genes.

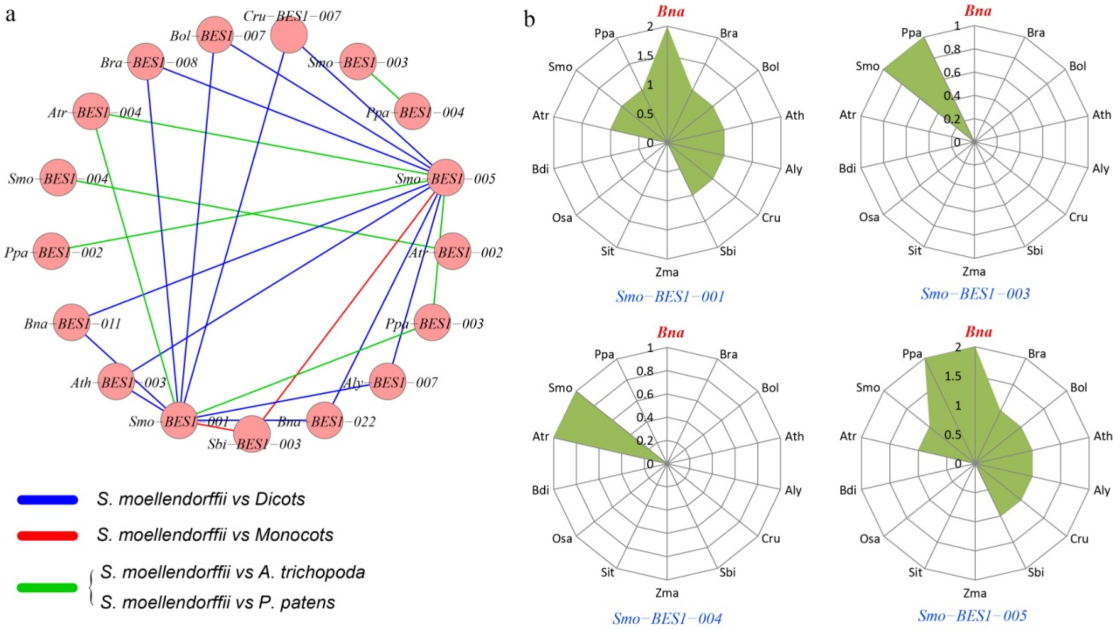

Figure S8. The analyses of orthologous *BES1* genes between *S. moellendorffii* and other examined species. (a) The interaction network constructed using the orthologous gene pairs between *S. moellendorffii* and other species. **(b)** The number of orthologous genes in each species with 4 *S. moellendorffii BES1* genes.

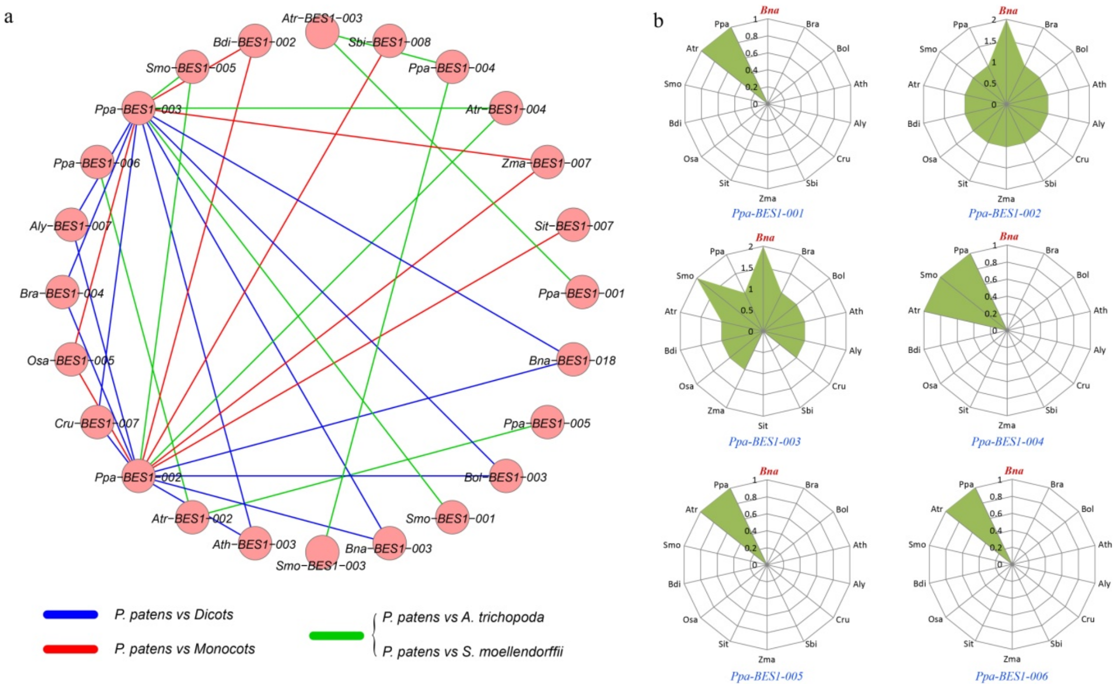

Figure S9. The analyses of orthologous *BES1* genes between *P. patens* and other examined species. (a) The interaction network constructed using the orthologous gene pairs between *P. patens* and other species. **(b)** The number of orthologous genes in each species with 6 *P. patens BES1* genes.

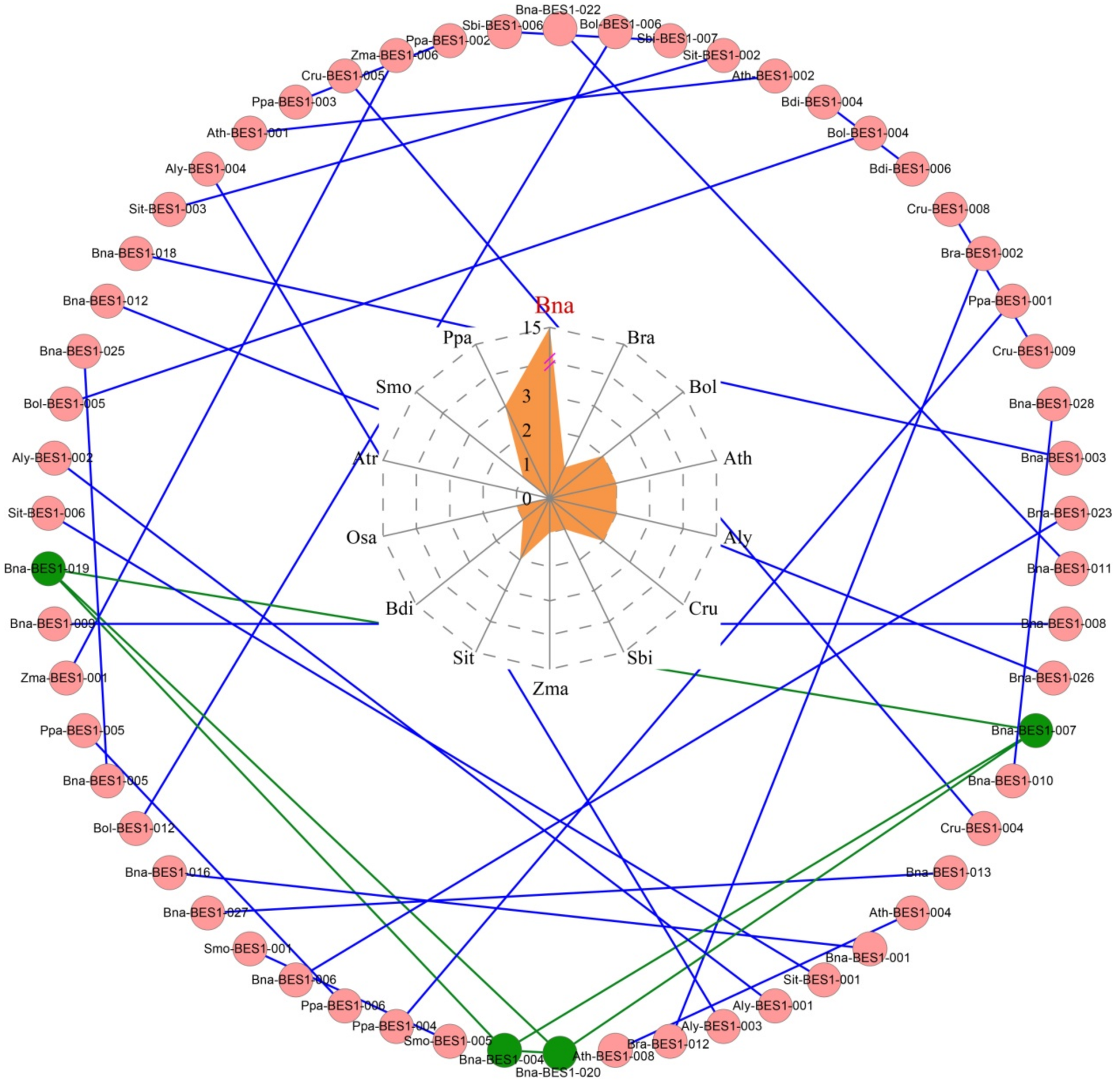

Figure S10. The analyses of paralogous *BES1* genes in each of the examined species. The green lines indicated the 4 *B. napus BES1* genes, which had more than 1 paralogous genes.

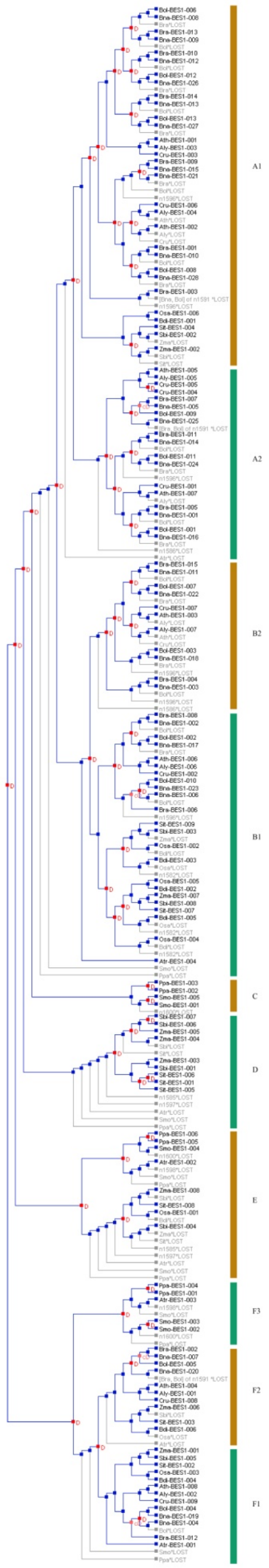

Figure S11. The reconstructed phylogenetic tree according to the duplication and losses of *BES1* genes at different stages of plant evolution.

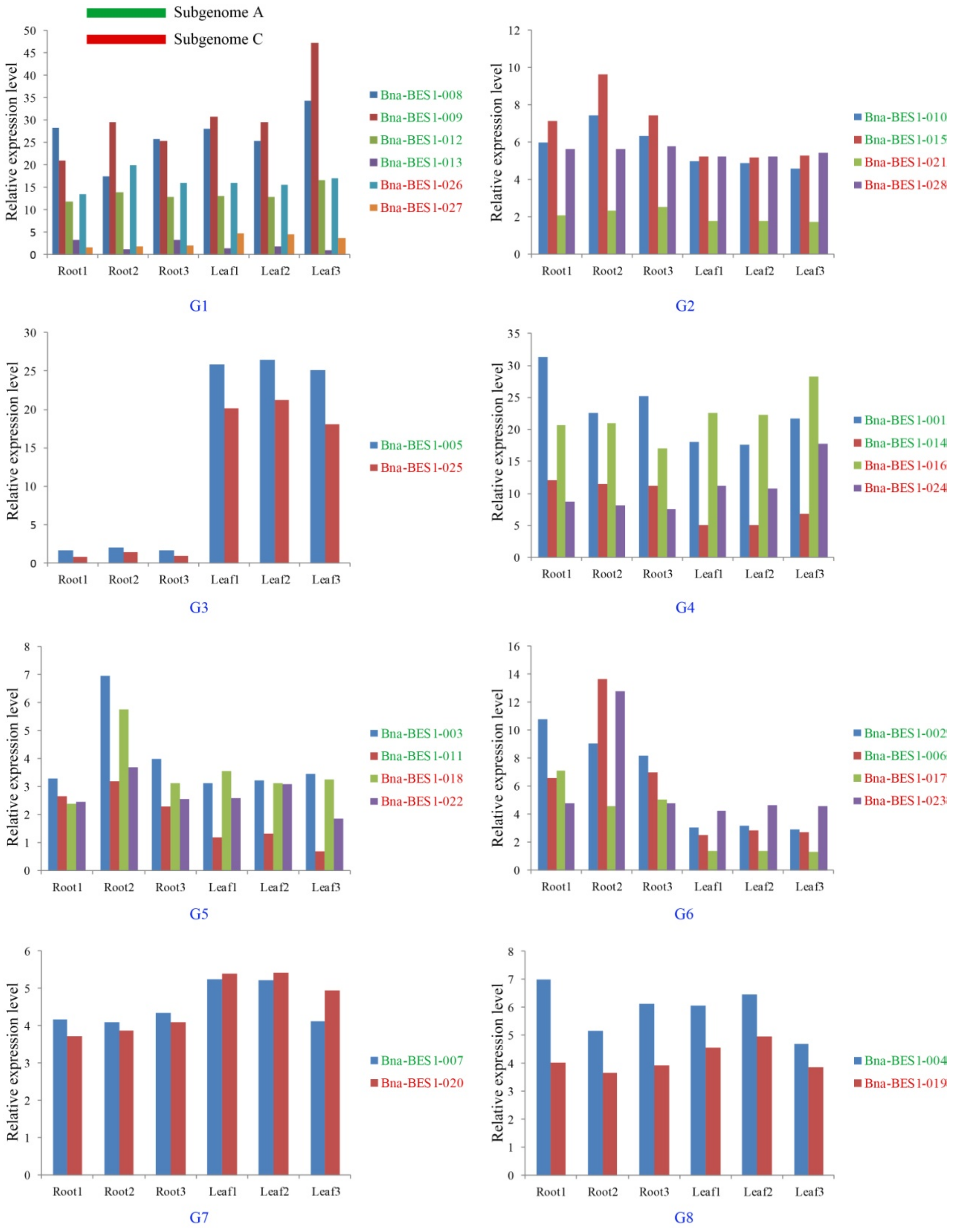

Figure S12. The histogram of *BES1* genes expression in root and leaf of *B. napus*. The genes marked with green located subgenome A, and red located subgenome C of *B. napus*.
